# Synthesis of highly active ETS-10-based titanosilicate for heterogeneously catalyzed transesterification of triglycerides

**DOI:** 10.3762/bjnano.10.200

**Published:** 2019-10-28

**Authors:** Muhammad A Zaheer, David Poppitz, Khavar Feyzullayeva, Marianne Wenzel, Jörg Matysik, Radomir Ljupkovic, Aleksandra Zarubica, Alexander A Karavaev, Andreas Pöppl, Roger Gläser, Muslim Dvoyashkin

**Affiliations:** 1 Institute of Chemical Technology, Universität Leipzig, Linnéstraße 3, 04103 Leipzig, Germany; 2 Institute of Analytical Chemistry, Universität Leipzig, Linnéstraße 3, 04103 Leipzig, Germany; 3 Faculty of Science and Mathematics, University of Nis, Visegradska 33, 18000 Nis, Serbia; 4 Gubkin Russian State University of Oil and Gas, Leninsky Prospekt 65, 119991 Moscow, Russia; 5 Felix-Bloch-Institut, Universität Leipzig, Linnéstraße 5, 04103 Leipzig, Germany

**Keywords:** CaO, diffusion, ETS-10, nuclear magnetic resonance (NMR), transesterification

## Abstract

In this contribution, the preparation of hierarchically structured ETS-10-based catalysts exhibiting notably higher activity in the conversion of triolein with methanol compared to microporous titanosilicate is presented. Triolein, together with its unsaturated analog trilinolein, represent the most prevalent triglycerides in oils. The introduction of mesopores by post-synthetic treatment with hydrogen peroxide and a subsequent calcination step results in the generation of an additional active surface with Brønsted basic sites becoming accessible for triolein and enhancing the rate of transesterification. The resulting catalyst exhibits a comparable triolein conversion (≈73%) after 4 h of reaction to CaO (≈76%), which is reportedly known to be highly active in the transesterification of triglycerides. In addition, while CaO showed a maximum conversion of 83% after 24 h, the ETS-10-based catalyst reached 100% after 8 h, revealing its higher stability compared to CaO. The following characteristics of the catalysts were experimentally addressed – crystal structure (X-ray diffraction, transmission electron microscopy), crystal shape and size (scanning electron microscopy, laser diffraction), textural properties (N_2_ sorption, Hg porosimetry), presence of hydroxyl groups and active sites (temperature-programmed desorption of NH_3_ and CO_2_, ^29^Si magic angle spinning nuclear magnetic resonance (NMR)), mesopore accessibility and diffusion coefficient of adsorbed triolein (pulsed field gradient NMR), pore interconnectivity (variable temperature and exchange spectroscopy experiments using hyperpolarized ^129^Xe NMR) and oxidation state of Ti atoms (electron paramagnetic resonance). The obtained results enabled the detailed understanding of the impact of the post-synthetic treatment applied to the ETS-10 titanosilicate with respect to the catalytic activity in the heterogeneously catalyzed transesterification of triglycerides.

## Introduction

One of the available solutions to address the world’s increasing energy consumption is the production of fatty acid methyl esters (FAMEs), also known as biodiesel, which are an alternative fuel similar to conventional diesel. They are usually produced from various triglycerides – components of vegetable or plant oils, animal fats and tallows [1]. Keeping in mind the critical aspect of food security (i.e., minimizing the use of edible feedstock types for fuel production) and the need for sustainability in the long run, recent developments in the use of algae as the feedstock have given the concepts of biofuel production renewed attention [2]. After several decades since the first reports on the usage of microalgae for biodiesel production [3–4], it has been determined that microalgal biofuels are promising candidates for the partial replacement of fossil fuels.

The production of microalgal biodiesel requires an efficient catalyst for initiation of the transesterification process that converts triglycerides into FAMEs. Other methods that do not require a catalyst, such as pyrolysis and utilization of supercritical fluid technology, are considered to be highly energy-intensive, inhibiting their practical implementation on the industrial scale [5].

The preparation and application of different types of catalysts for homogeneous, heterogeneous, and even enzymatic transesterification processes have been extensively investigated [6]. However, the most commonly used commercial process for biodiesel production is the homogenous transesterification of triglycerides with methanol in the presence of sodium hydroxide (NaOH) or potassium hydroxide (KOH), which is still in use on an industrial scale. Along with the obvious advantages of being highly active and relatively inexpensive (depending however on the number of washing steps and possible need for neutralization), the downsides of alkali hydroxides are long known – difficulties in separation of the K^+^/Na^+^ traces from the product, higher catalyst consumption compared to the solid ones and low reusability [7]. On the other hand, the reported solid catalysts can potentially resolve issues associated with catalysts used in the homogeneous process, that is, they can be prepared with a desired particle size for separation needs and can be reused. The main drawbacks are their lower activity compared to the catalysts used in homogeneous solutions (often associated with mass transfer issues [7]) and the leaching of the active phase (if supported catalysts are used [8]).

Thus, the search continues for a new generation of catalysts that ideally combine the advantages of both types of catalysts used in the homo- and heterogeneously catalyzed reactions [9–12]. In the context of biodiesel production via the transesterification of triglycerides with methanol, the challenge is to prepare a catalyst possessing the following characteristics: i) large particle size for convenient separation, ii) accessibility of the active sites for reactants, iii) minimized diffusion limitations for reaching effectiveness factors close to unity, iv) leaching resistance, v) activity at moderate temperatures (e.g., at the boiling point of methanol) being ideally comparable to KOH/NaOH catalysts and vi) stability for multiple reuse. Our approach to address this challenge is based on the preparation of bulk catalysts that are reported to be active in the transesterification reaction together with further (nano)structural modification aimed at enhancing accessibility to the active sites and improving the mass transfer characteristics for efficient reactant supply and product removal [13].

Amongst the prospective solid catalysts designed for transesterification reactions, such as calcium [14] and other metal oxides [15], metal–organic frameworks (MOFs) [10], silica-supported catalysts [16], biochar [17] and other biomass-derived catalysts [18], zeolites and molecular sieves [19–20] offer a combination of the possibility for the pore network modification (e.g., as a result of a post-synthetic treatment) and high stability (e.g., compared to MOFs) with active sites being part of a framework.

**Figure 1:**
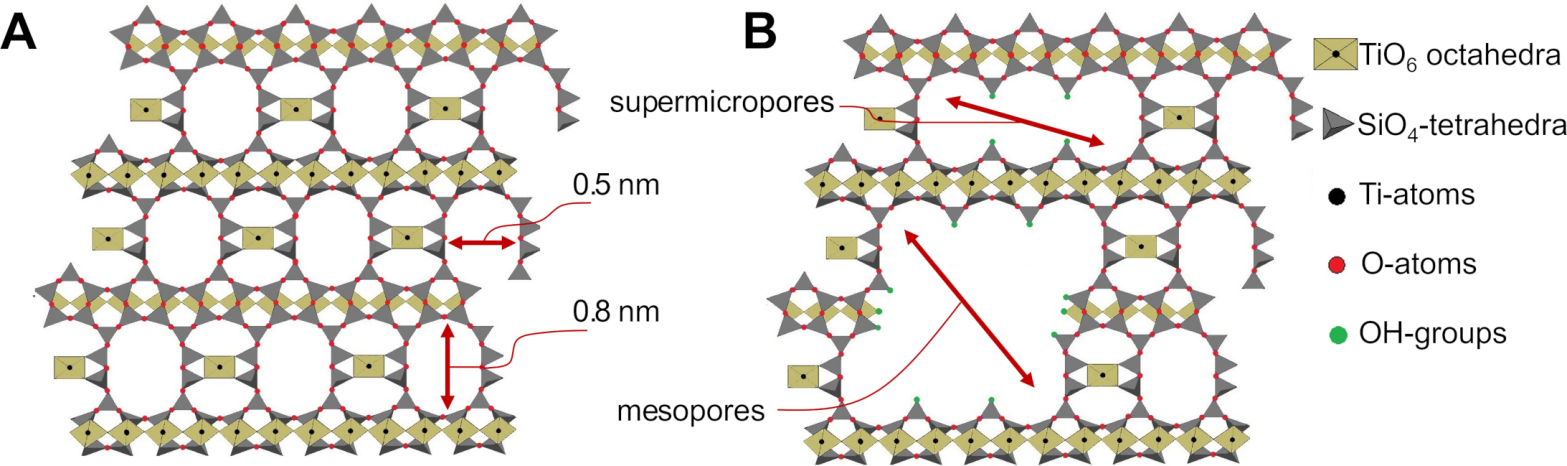
One of the polymorphs of the microporous ETS-10 consisting of TiO_6_ octahedra and SiO_4_ tetrahedra sharing oxygen atoms (A). Possible framework modification with present super-micropores and mesopores that might result from the post-synthetic treatment of ETS-10 with H_2_O_2_ aqueous solution according to [26] (B).

The microporous, titanosilicate ETS-10 catalyst was found to be one of the most active catalysts amongst the crystalline microporous molecular sieves (such as, e.g., zeolites) reported for the transesterification of triglycerides with methanol [20]. Its crystal structure is built up from orthogonal TiO_6_ octahedra and SiO_4_ tetrahedra sharing oxygen atoms and forming a three-dimensional interconnected pore system [21] consisting of channels with cross-sectional dimensions of 0.8 × 0.5 nm [22–23] (Figure 1A). Each Ti atom in a six-coordinated state bears two negative charges, which can be balanced by Na^+^ or K^+^ cations according to the following stoichiometry (Na,K)_2_TiSi_5_O_13_ [22]. The high activity of ETS-10 in the transesterification reactions compared to zeolites and other molecular sieves originates presumably from its pronounced Brønsted basicity of the shared oxygen atoms [20]. For example, it has been reported that the parent ETS-10 catalyst is approximately four times more basic than the NaX zeolite based on acetone selectivity in the conversion of isopropanol to acetone and propene [24]. However, due to its microporous nature, to the best of our knowledge, in all reported studies on the transesterification of triglycerides (single-component or in mixtures/oils) for biodiesel production, the reaction took place solely on the outer crystal surface. The smallest triglyceride, triacetin, used in these studies, has a reported critical diameter of ≈1 nm [25], which appears to be too large to diffuse into the micropores.

Several strategies have been attempted for a post-synthetic modification of the ETS-10: an ion-exchange method [27], acidic treatment [28–29], and treatment with hydrogen peroxide (H_2_O_2_) [26,30–31]. The latter approach resulted in a notable pore network modification by introducing larger micropores (≈1–2 nm) and mesopores (≈5–30 nm) in a controllable way, for example, by using different concentrations of the H_2_O_2_ solution and treatment times (Figure 1B). Herewith, the crystallinity and mechanical stability of the materials were well-preserved despite the experienced desilication and detitanation. This approach was followed in the present study to prepare hierarchically structured ETS-10 crystals (i.e., containing interconnected micro- and mesopores). In general, the micropores of the hierarchical catalysts mostly contribute to the catalytic process, while the function of the larger mesopores is the promotion of the reactant supply and product removal. However, for bulky triglycerides, the micropores are not expected to be accessible. In such a case, the mesopores will fulfil the task of providing access to the catalytically active sites located within them.

In the present contribution, we report the successful preparation of large crystallite (≈30 μm) ETS-10 titanosilicates with improved active site accessibility (achieved by post-synthetic treatment) for efficient transesterification of triglycerides into biodiesel. A triolein has been selected as the triglyceride due to its prevalence in, for example, microalgal oils typically used in production of 3rd and 4th generation biodiesel. Additionally, CaO catalysts, known to be highly active in the transesterification process, were prepared as references for comparison of the catalytic activity under identical reaction conditions. Prior to catalytic tests, the prepared CaO- and titanosilicate catalysts were characterized to obtain quantitative information on properties such as crystal structure by X-ray diffraction (XRD), crystal size by laser diffraction, crystal morphology by scanning electron microscopy (SEM) and transmission electron microscopy (TEM), pore width by N_2_ sorption and Hg intrusion, acid and basic site density by NH_3_ and CO_2_ temperature-programmed desorption (TPD), presence of hydroxyl groups by ^29^Si magic angle spinning nuclear magnetic resonance (MAS NMR), pore interconnectivity by hyperpolarized (HP) ^129^Xe NMR, pore accessibility for triolein by ^1^H pulsed field gradient (PFG) NMR, state of the Ti atoms before and after the treatment of titanosilicates by electron paramagnetic resonance (EPR) and thermal stability of the crystals by differential thermal analysis (DTA).

## Results and Discussion

### Structure characterization of an as-synthesized ETS-10 titanosilicate

**Figure 2:**
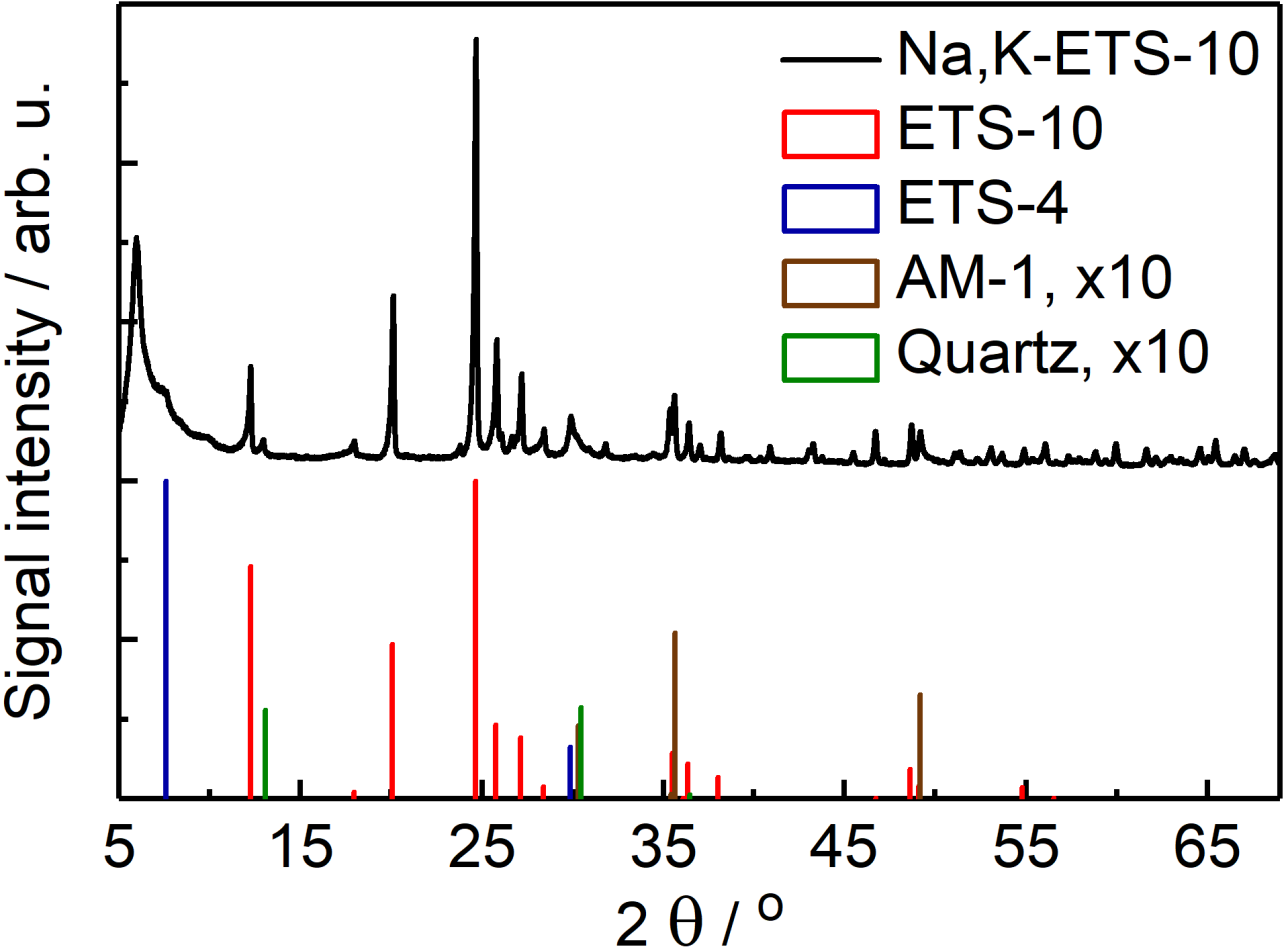
X-ray diffractograms of the as-synthesized ETS-10 material (Na,K-ETS-10, top) and of the reference reflections of ETS-10, ETS-4, AM-1 and quartz according to [33–36]. Signals of AM-1 and quartz are 10-fold amplified for visibility.

**Figure 3:**
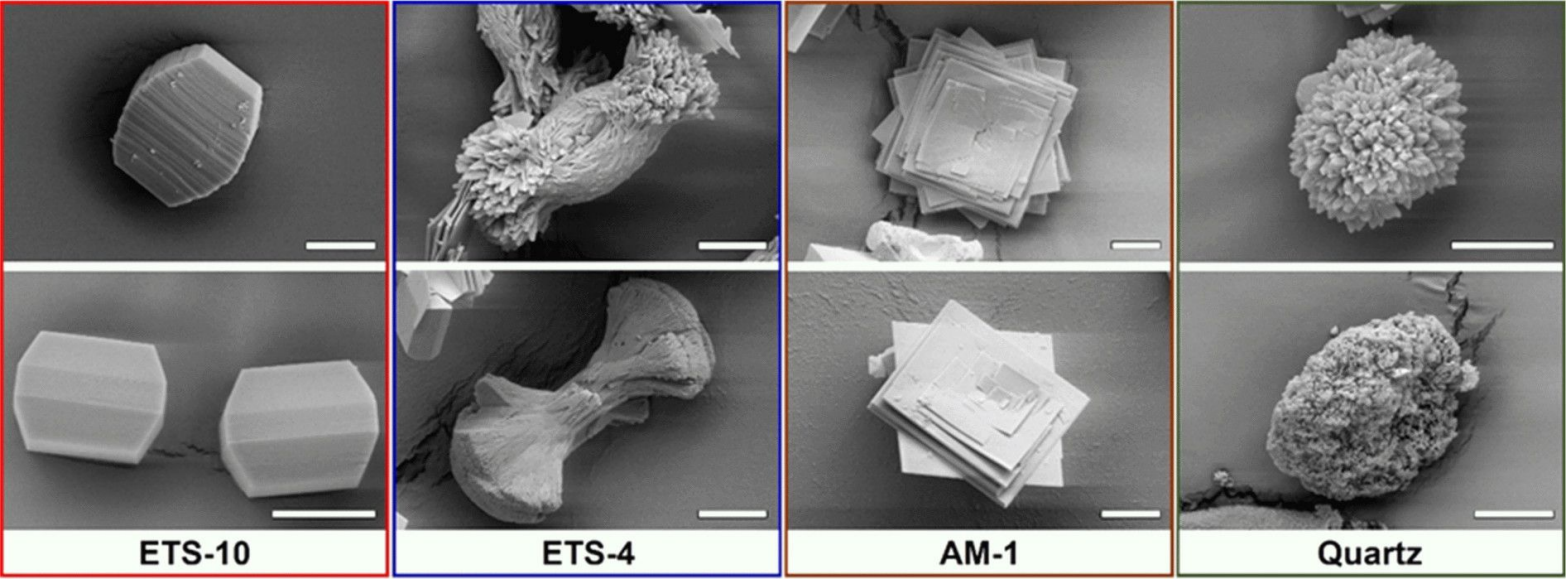
SEM micrographs of selected crystals representing different types of phases formed during the synthesis of titanosilicates. The assignment of each crystal shape to the corresponding phase was done based on the Si/Ti (for titanosilicates) and Si/O (for quartz) ratios obtained from the EDX analysis (see Table S8 in Supporting Information File 1). The scale bar in all images is 10 μm.

Figure 2 represents XRD data obtained on an as-synthesized ETS-10 (Na,K-ETS-10) plotted together with reference diffractograms of the phases that can be formed as by-products during the synthesis of ETS-10 [30,32]. Thus, in addition to the prevalent reflections being characteristic for ETS-10, smaller quantities of ETS-4, AM-1 and quartz were detected (see also SEM data in Figure 3). The corresponding reflections at angles of 2θ are seen at, for example, 29.9° and 7.6° from ETS-4, 35.6° and 49.2° from AM-1, and 13.1° and 30.4° from quartz. The quantification of the presence of each phase in the prepared batch, as well as the results of the studies aimed at minimization of the amount of quartz and alternative titanosilicate phases employed during the synthesis of ETS-10 are described in section S6 of Supporting Information File 1. The presence of sharp peaks in the diffractogram (e.g., 0.15° FWHM for the peak at 24.63°) reflects the high crystallinity of the prepared ETS-10 material.

**Figure 4:**
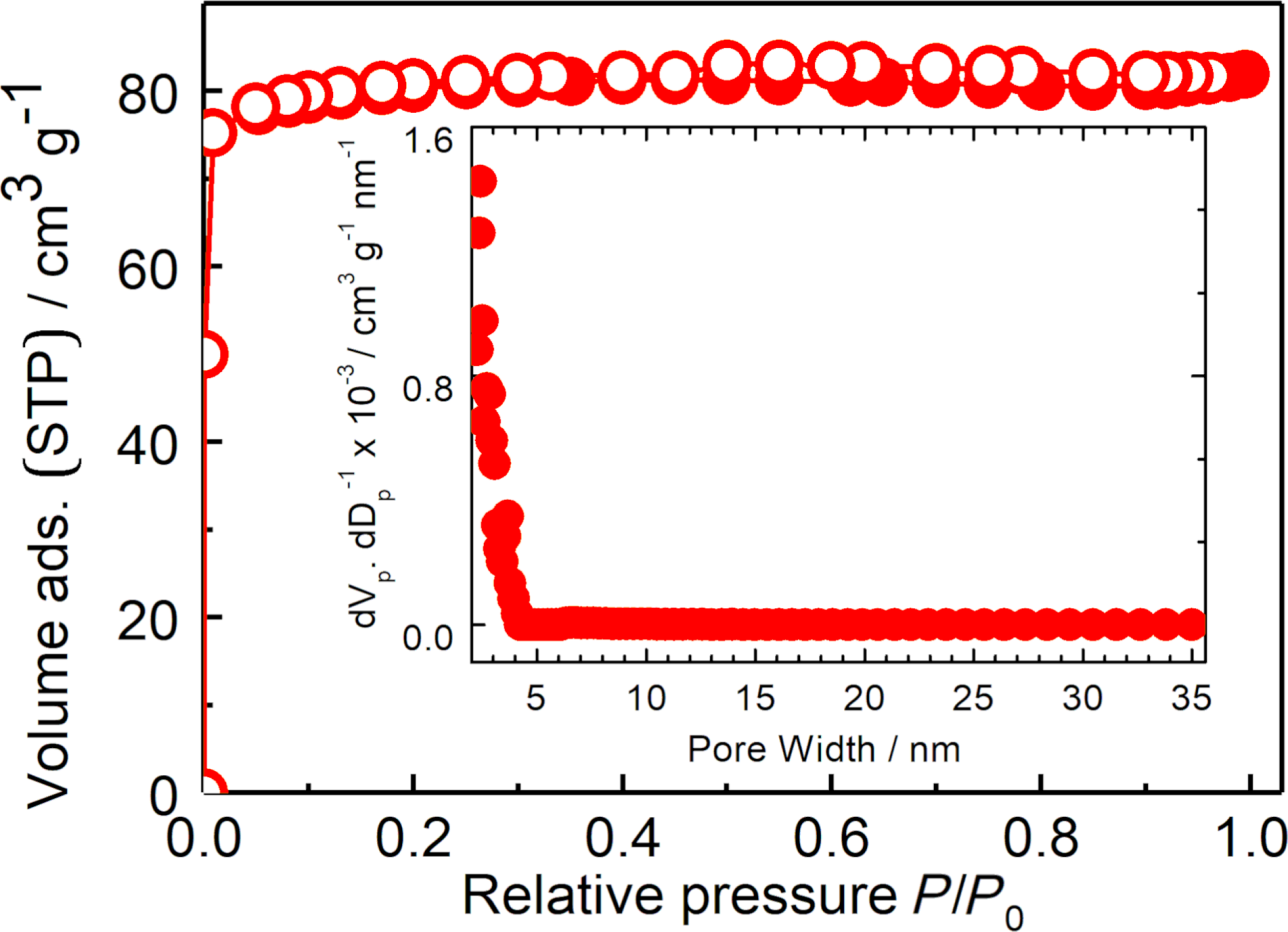
Nitrogen adsorption (solid circles) and desorption (open circles) isotherms of the as-synthesized Na,K-ETS-10. The inset shows the calculated pore width distribution according to the NLDFT.

**Figure 5:**
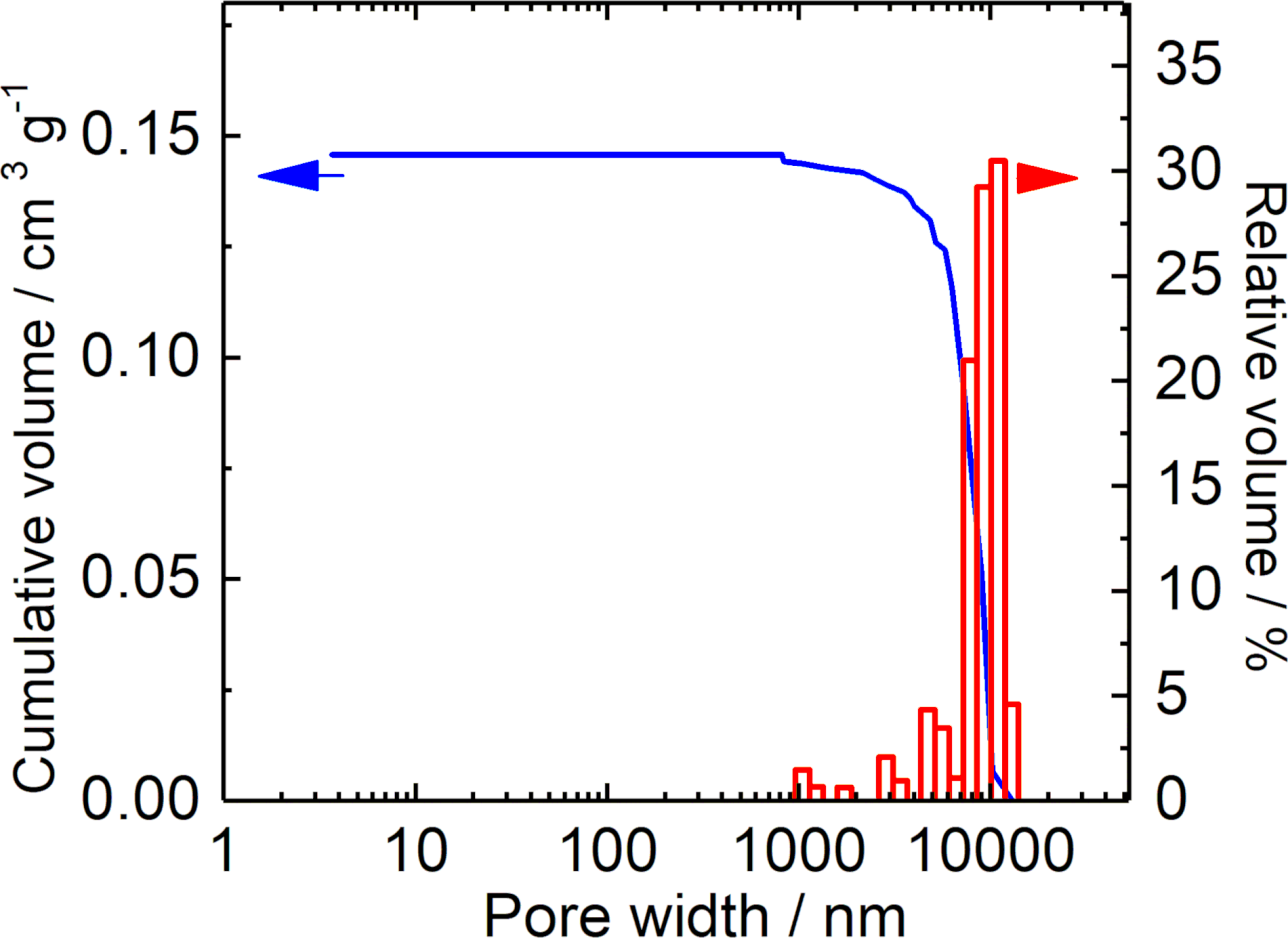
Hg intrusion data of the as-synthesized Na,K-ETS-10 material.

Data of the textural analysis by N_2_ sorption into the Na,K-ETS-10 sample is presented in Figure 4. The isotherms exhibit the Type I shape [37] (revealing the presence of solely micropores) and have a specific surface area of 257 m^2^ g^−1^ and micropore volume of 0.110 cm^3^ g^−1^. The pore width distribution calculated from the adsorption branches of the isotherm using the NLDFT method is presented in the inset of Figure 4. The same procedure was applied for the characterization of other (treated) titanosilicates. The Hg intrusion data shown in Figure 5 demonstrate the absence of pores in the range from 5 nm to ≈1 μm, suggesting that the amount of possible defects in the crystals is negligibly small. The textural data of all titanosilicates prepared for catalytic studies are summarized in Table 1.

**Table 1:** Textural characteristics of the titanosilicates from the results of the N_2_ sorption experiments.

Material	BET specific surface area / m^2^ g^−1^	Specific micropore volume^a^ / cm^3^ g^−1^	Specific mesopore volume / cm^3^ g^−1^	Specific total pore volume^b^ / cm^3^ g^−1^

Na,K-ETS-10	257	0.110	0.017	0.127
P-ETS-10/30	262	0.110	0.022	0.132
P-ETS-10/45	282	0.111	0.070	0.181
P-ETS-10/60	291	0.131	0.098	0.229
C-P-ETS-10/60	235	0.105	0.084	0.189

^a^Micropore volume was calculated by t-plot at a relative pressure range of *P*/*P*_0_ = 0.15–0.5; ^b^Total pore volume was calculated at *P*/*P*_0_ = 0.99 according to the Gurvich rule, and the mesopore volume is calculated by subtracting the micropore volume from the total pore volume.

**Figure 6:**
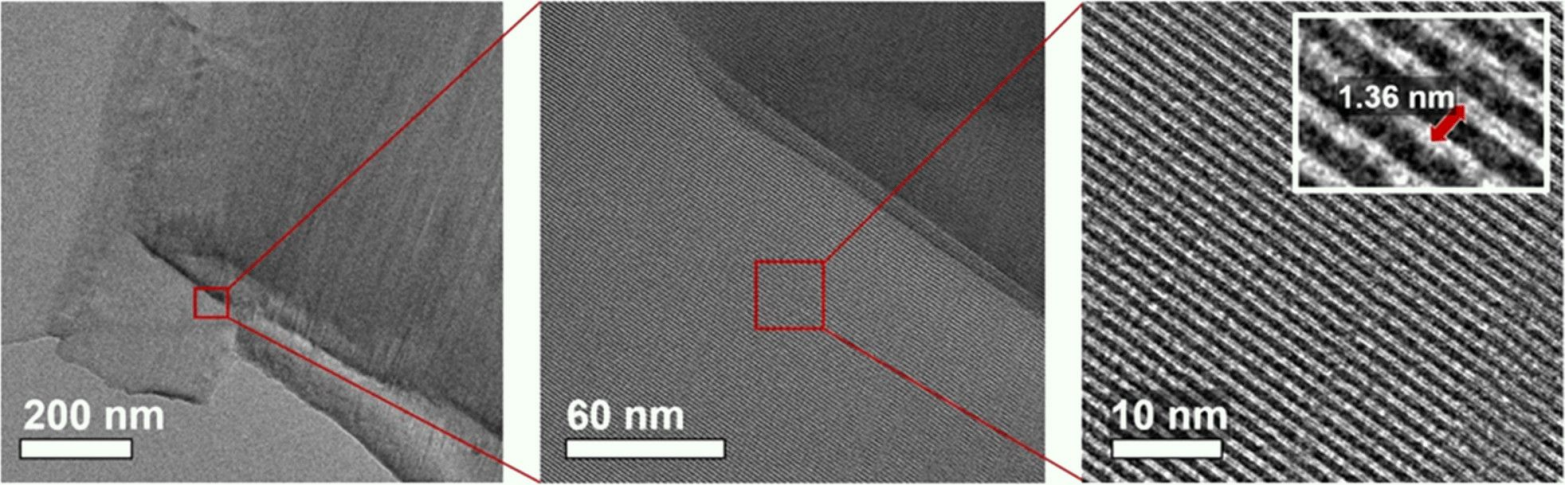
TEM micrographs for the Na,K-ETS-10 material obtained with different magnifications demonstrating a highly ordered ETS-10 crystal structure. Other images obtained with different ETS-10 crystals can be found in section S9 of Supporting Information File 1.

The microporous nature and high crystallinity of Na,K-ETS-10 is further confirmed by TEM (Figure 6) demonstrating high order of the titanosilicate framework with parallel Ti nanowires showing no visible defects on the length scale of hundreds of nanometers. The spacing between adjacent nanowires showed good agreement with literature data of 1.36 nm [38].

### Impact of H_2_O_2_ treatment on crystallinity, textural characteristics, surface chemistry and pore interconnectivity of ETS-10 titanosilicates

**Figure 7:**
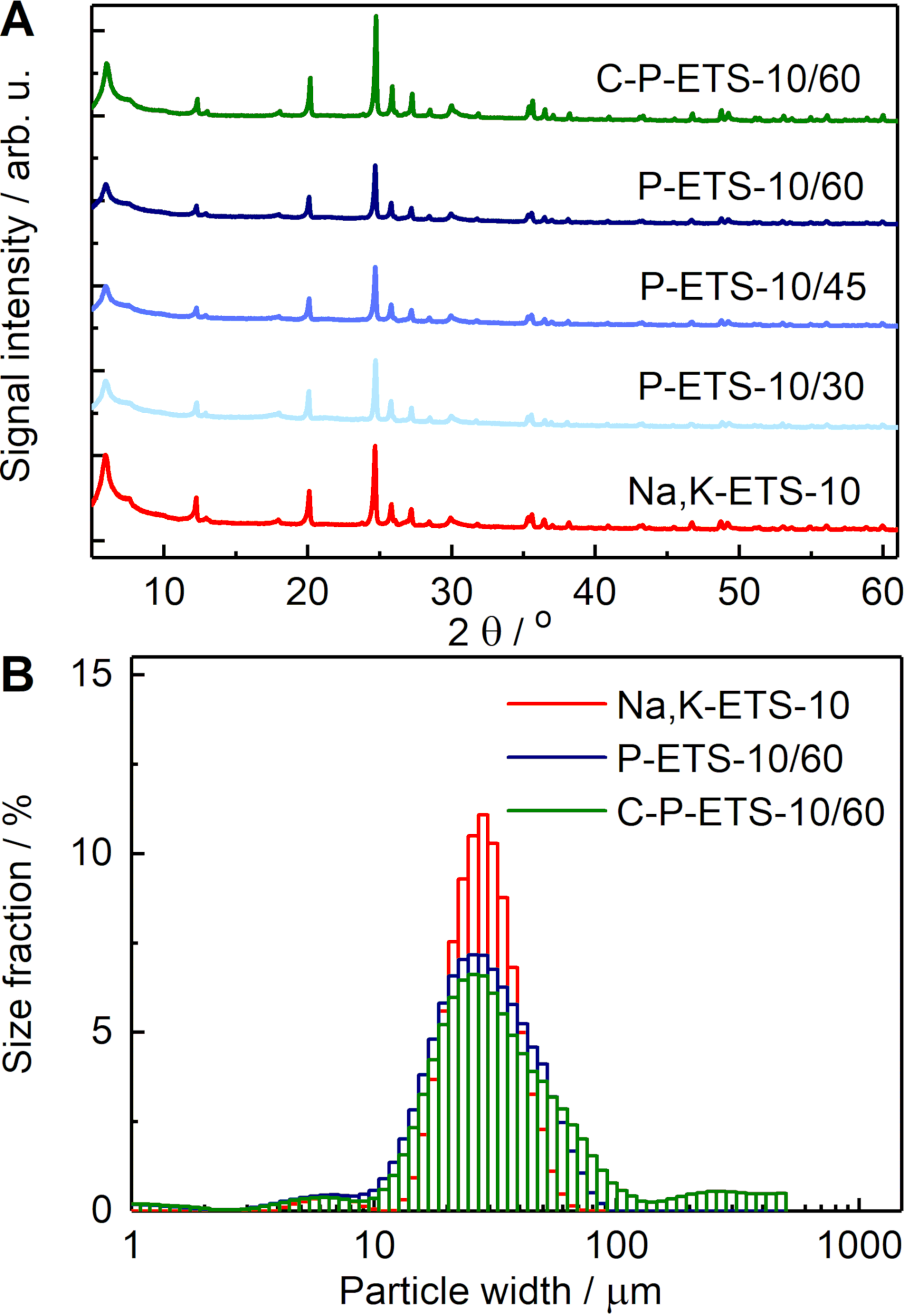
(A) XRD data of post-synthetically treated titanosilicates. The calculated crystallinity decreased to ≈70% for P-ETS-10/30, P-ETS-10/45 and P-ETS-10/60, and was restored to 100% for C-P-ETS-10/60 (assuming the parent material is 100%). (B) Particle size distribution obtained by laser diffraction for Na,K-ETS-10 (red), P-ETS-10/60 (blue) and C-P-ETS 10/60 (green).

**Figure 8:**
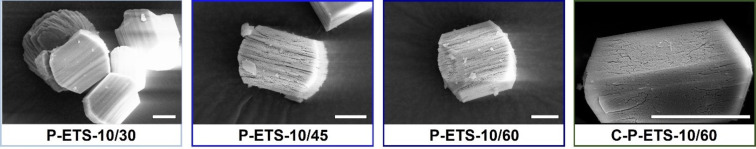
SEM micrographs of the titanosilicates treated with H_2_O_2_ for 30, 45 and 60 min (P-ETS-10/30, P-ETS-10/45, P-ETS-10/60). P-ETS-10/60 additionally underwent calcination at 873 K for 6 h. The scale bar in all images is 10 μm.

The XRD data of treated samples in Figure 7A demonstrate that the treatment of titanosilicates with H_2_O_2_ and subsequent calcination does not notably affect the crystallinity, which is fully restored after the calcination (see Table S10 of Supporting Information File 1). However, partial removal of the framework has led to the appearance of crack-looking defects on the surface of ETS-10, less noticeable on the crystals treated for 30 min, but more pronounced for longer treatment times (Figure 8). Despite these defects, the particle size distribution probed by laser diffraction revealed minor changes suggesting that treatment neither leads to dissolution of smaller particles, nor to detectable fractioning of the larger ones (Figure 7B).

**Figure 9:**
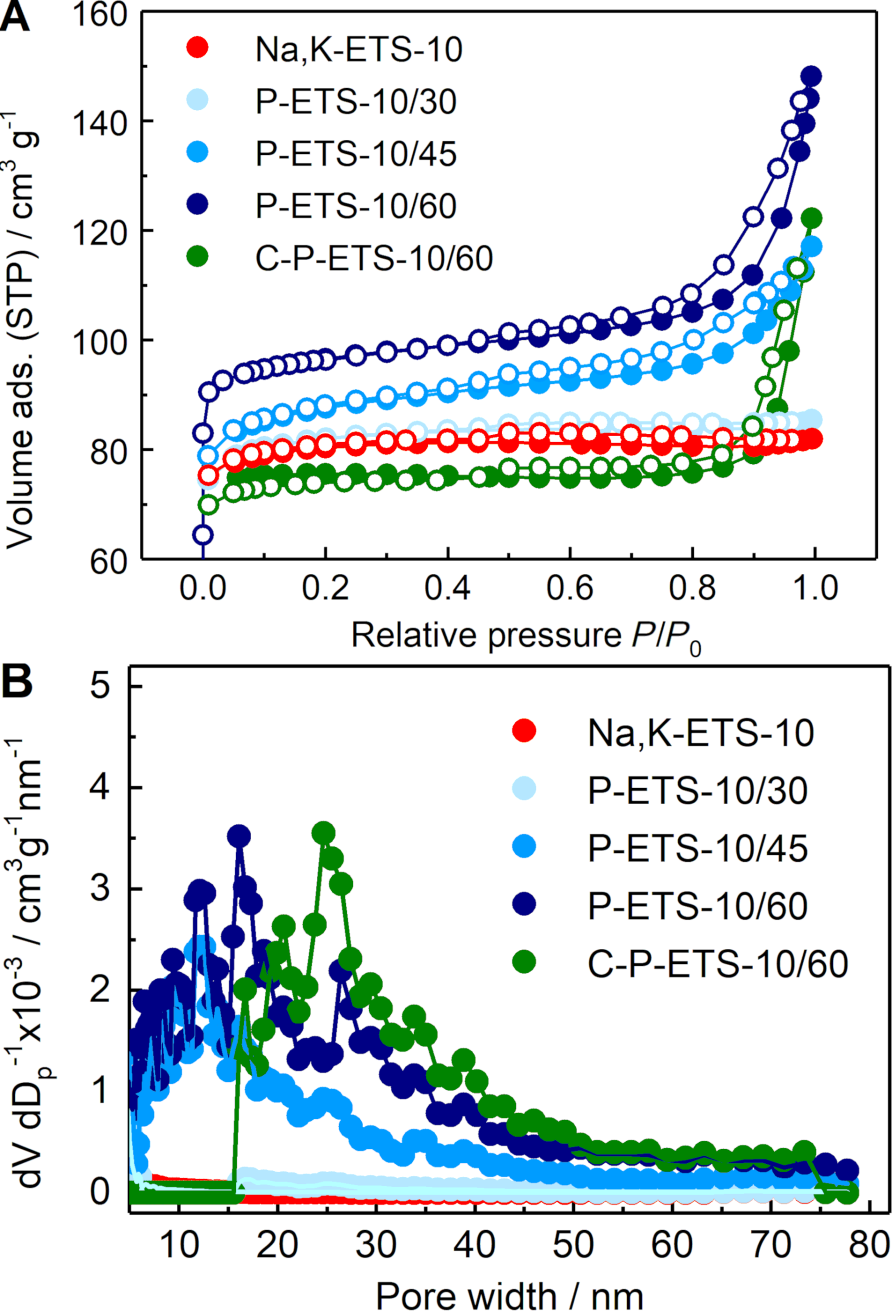
(A) Nitrogen adsorption and desorption isotherms of titanosilicates treated with H_2_O_2_ for 30 min (light blue), 45 min (blue) and 60 min (dark blue) and of the latter after calcination (green). The sorption isotherm of as-synthesized Na,K-ETS-10 (red) is shown for comparison. (B) Calculated pore width distributions using the NLDFT.

**Figure 10:**
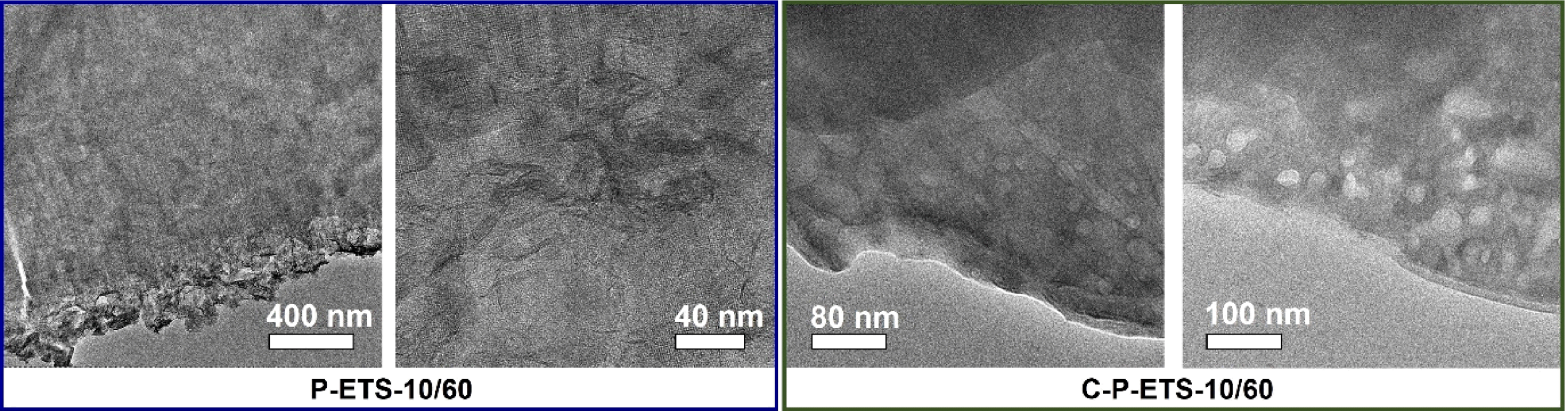
TEM images of the selected crystals of P-ETS-10/60 and C-P-ETS 10/60 demonstrating the presence of mesopores that appeared after treatment.

The N_2_ sorption isotherms revealed an increasing impact of treatment on the textural properties of the titanosilicates with longer treatment time (Figure 9A) – the total pore volume increased from 0.127 cm^3^ g^−1^ for Na,K-ETS-10 to 0.132, 0.181 and 0.229 cm^3^ g^−1^ after 30, 45 and 60 min of contact time with H_2_O_2_, respectively. This change results from the contribution of mesopores in the range of 5–40 nm formed during the treatment (see Figure 9B and Table 1). This is consistent with the TEM images of treated samples with clear identification of mesopores presented in Figure 10. It is worth noting that the appearance of mesopores after treatment has led to the change of the isotherm shape from type I (typical for microporous solids) to a combination of types I and IV, as is expected for micro–mesoporous materials [37].

It was suggested that exposure of the ETS-10 framework to H_2_O_2_ solution leads to its partial removal either by the disruption of Ti–O–Ti chains and partial leaching of Ti atoms [26] or by microexplosions if elevated temperatures are used [39]. At the same time, the measured specific micropore volume either does not change (for 30 and 45 min of treatment time) or slightly increases from 0.110 cm^3^ g^−1^ for the parent titanosilicate to 0.131 cm^3^ g^−1^ after 60 min of treatment. The application of calcination has led to a slightly lower mesopore volume for P-ETS-10/60 (0.098 cm^3^ g^−1^) and C-P-ETS-10/60 (0.084 cm^3^ g^−1^) and to micropore volumes of 0.131 cm^3^ g^−1^ and 0.105 cm^3^ g^−1^, respectively. Referring to the XRD data, which reveals that the structure of the ETS-10 material after calcination remains intact (also preserving high crystallinity), it is not expected that the micropore volume will notably decrease. This observation can be attributed to the different interactions of the titanosilicate surface with the quadrupole momentum of N_2_ for P-ETS-10/60 and C-P-ETS-10/60. In such a case, the precise analysis of the microporosity by N_2_ is difficult and the use of Ar instead is recommended [26,40].

**Figure 11:**
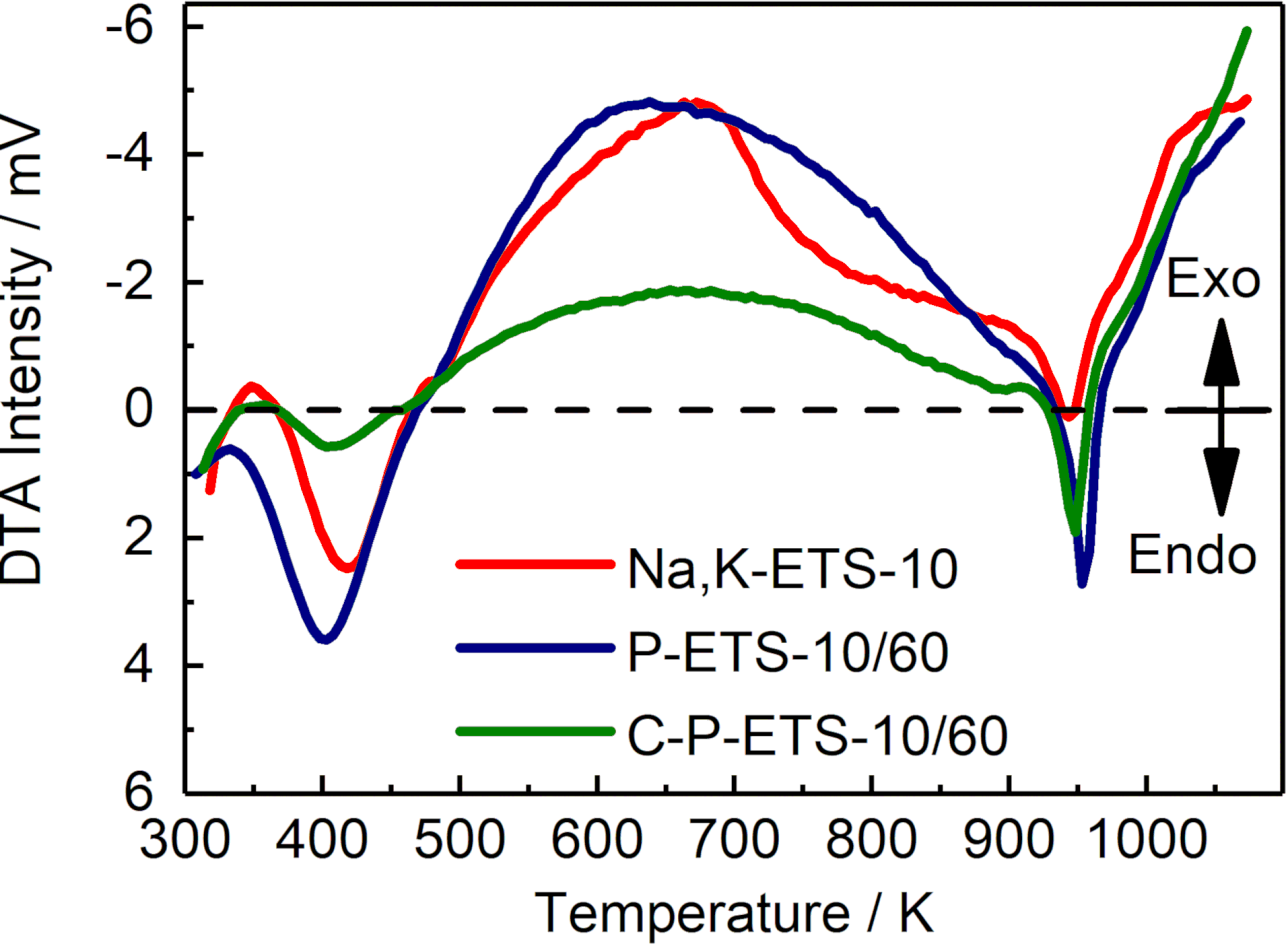
Differential thermal analysis of the Na,K-ETS-10 (red), P-ETS-10/60 (blue) and C-P-ETS-10/60 (green) materials. The dashed line separates regions of exothermic (exo) and endothermic (endo) processes.

The calcination of P-ETS-10/60 has led to a visible decrease of the adsorbed volume in the range of 5–15 nm and to a shift of the maximum of the pore width distribution towards larger values. One possible explanation of this observation is the appearance of additional structural defects (to those resulting from the H_2_O_2_ treatment) due to exposure of the material to high temperatures (i.e., 873 K) for 6 h. Such thermal treatment might cause cracks in part of the framework, for example, separating two adjacent mesopores, leading to merging into a larger void. It was reported that the ETS-10 framework can collapse at temperatures above 920 K [41]. To determine this critical temperature, which might vary for titanosilicates prepared under different conditions, DTA was conducted where a critical temperature of ≈950 K was determined (Figure 11). This is only 77 K higher than the calcination temperature employed, which might be sufficient to introduce such cracks upon heating. On the other hand, this temperature difference seems to be large enough to prevent destruction of the framework during the calcination. The low-temperature minimum (first weight loss) is due to dehydration, while the exothermic part in the range 500–900 K can be attributed to the burning-out of the surface hydroxyl groups [42–43].

Since it is expected that the active sites located inside the micropores of the catalysts are not accessible for bulky triolein, their contribution in the transesterification process is expected to be negligibly small. Thus, further analysis of the textural characteristics of the micropores by Ar was not conducted. The 60 min titanosilicate-treated P-ETS-10/60 and its calcined counterpart C-P-ETS-10/60 were selected for further characterization and catalytic studies as those exhibiting the highest mesopore volume.

Another limitation of using N_2_ is the analysis of the macropores (>50 nm) [37], for which the Hg porosimetry was considered. The obtained data show a significant fraction of voids in the range of ≈1–20 μm (see Figure S11 of Supporting Information File 1), which was assigned to the interparticle spaces that can be filled by Hg after applying pressure. This is consistent with the similar crystal sizes measured by laser diffraction (Figure 7B). As expected, below 1 μm no Hg intrusion can be seen for the microporous Na,K-ETS-10, while for P-ETS-10/60 and C-P-ETS 10/60, a visible fraction in the range of mesopores and macropores is detected. The obtained specific mesopore volumes were 0.114 cm^3^ g^−1^ and 0.069 cm^3^ g^−1^ for P-ETS-10/60 and C-P-ETS 10/60, respectively. This appears to be in good agreement with the corresponding values obtained by N_2_ sorption (Table 1, column 3). The specific surface area from the Hg intrusion was 0.1 m^2^ g^−1^ for Na,K-ETS-10, 52.6 m^2^ g^−1^ for P-ETS-10/60, and 24.2 m^2^ g^−1^ for P-ETS-10/60. In the conducted Hg porosimetry experiments, in contrast to the N_2_ sorption, the micropores were not reachable for Hg, while the macropores did contribute to the measured surface area.

### Characterization of acidity and basicity of the ETS-10-based titanosilicates

**Figure 12:**
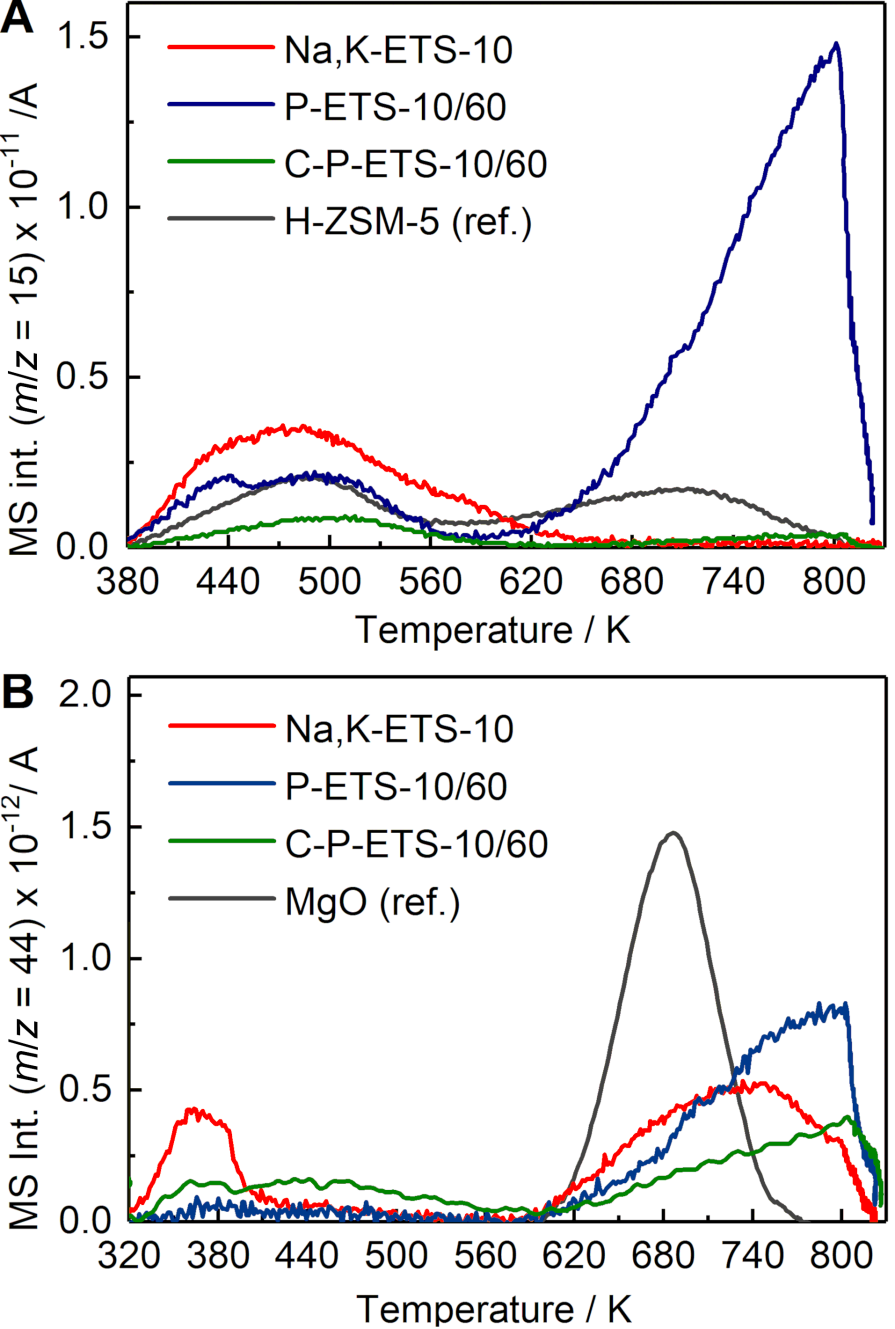
Temperature-programmed desorption of NH_3_ (A) and CO_2_ (B) in Na,K-ETS-10 (red), P-ETS-10/60 (blue) and C-P-ETS 10/60 (green) samples. For comparison, NH_3_ desorption from the H-ZSM-5 zeolite with Si/Al = 27 and a known acid site density used as a reference is presented by the grey line. The curve of MgO was used as a reference in B.

NH_3_ and CO_2_ were used as probe molecules in TPD studies to determine the surface acid/basic site density of the prepared titanosilicates. Desorption of NH_3_ presented in Figure 12A occurs in one (Na,K-ETS-10) or two (P-ETS-10/60, C-P-ETS-10/60) distinguishable steps. According to [44], the range ≈380–600 K represents temperatures of NH_3_ desorption from the weak Lewis acid sites, for example, Na^+^ or K^+^ cations, being more pronounced in Na,K-ETS-10, which is partially due to the higher amount of present cations compared to the treated titanosilicates (Table 2, last column). In all materials this desorption step extends over ≈200 K, probably due to the presence of different cationic sites (I–V) near the titanate chains, which are described in [45] in detail. NH_3_ preferentially coordinates near the type V cations located at the top of the 12 ring pore near a titanate chain. After treatment, these vacancies are partially removed due to the detitanation process. This can additionally contribute to the decrease in the observed intensities of NH_3_ desorption below 600 K for treated samples compared to Na,K-ETS-10. At temperatures above 600 K, highly pronounced NH_3_ desorption, presumably from the surface hydroxyl groups being strong Brønsted acid sites, appeared as a result of the H_2_O_2_ treatment. The calcination process leads to dehydroxylation of the surface [42], which notably reduces the NH_3_ desorption for C-P-ETS-10/60 above 600 K as compared to P-ETS-10/60. Table 2 summarizes the calculated values of the surface acid/basic site density and intrinsic acid/basic adsorption capacity of the titanosilicates, as calculated by

[1] $$\frac{\text{Surface acid (basic) site density [}\mu {\text{mol g}}^{-1}\text{]}}{{\text{Specific surface area [m}}^{2}{\text{ g}}^{-1}\text{]}}$$

**Table 2:** NH_3_ TPD, CO_2_ TPD, and ICP OES data for Na,K-ETS-10, P-ETS-10/60, and C-P-ETS-10/60. n.d. – no data.

Sample	Surface acid site density / μmol g^−1^	Surface basic site density / μmol g^−1^	Specific surface area / m^2^ g^−1^	Intrinsic acid adsorption capacity^a^ / μmol m^−2^	Intrinsic base adsorption capacity^a^ / μmol m^−2^	wt % of elemental Ti, Na, and K^b^

Na,K-ETS-10	1324	136	257	5.15	0.52	9.7, 9.0, 0.7
P-ETS-10/60	3955	145	291	13.59	0.49	11.0, 7.0, 0.9
C-P-ETS-10/60	359	92	239	1.50	0.38	9.8, 7.0 1.1
H-ZSM-5^c^	1186	n.d.	366	3.24	n.d.	–
MgO^c^	n.d.	959	15	n.d.	63.9	–

^a^Calculated using Equation 1; ^b^Experimental error in the ICP OES analysis is ≈1 wt %; ^c^H-ZSM-5 and MgO were used as references with known surface acid and basic site density.

The results of the CO_2_ TPD in the parent and treated titanosilicates are presented in Figure 12B. The TPD curve of Na,K-ETS-10 reveals two distinct peaks in the range ≈320–420 K and above 600 K. The desorption in the low-temperature range is presumably due to Na^+^ or K^+^ cations and weakly basic surface hydroxyl groups [46–47], i.e., the H-bridged Ti–OH–Si (see also Figure 3 of [31]). As a result of the post-synthetic treatment by H_2_O_2_, a decrease of the desorption peak in this temperature range for P-ETS-10/60 might be due to the partial removal of the cations (decrease of the Na wt % from 9 to 7 after treatment with H_2_O_2_, see Table 2 (last column)) and of the H-bridged surface hydroxyl groups, as is also evident from the diffuse reflectance infrared Fourier transform (DRIFT) study in [31]. After calcination, the peak below 600 K slightly increases and becomes broad.

The high-temperature range for all three titanosilicates is characterized by notable desorption from the strong Brønsted basic sites. For Na,K-ETS-10, these are the oxygen atoms shared between Si and Ti and bearing uncompensated negative charge (O^−^), and the isolated hydroxyl groups forming the hydrogen carbonate species HO–CO_2_^−^ [48]. It is worth noting that probably only those O^−^ Brønsted sites are sensed by CO_2_ which are located between the 12-membered ring pore and the Ti–O–Ti nanorods. The treatment with H_2_O_2_ leads to higher CO_2_ desorption above 600 K, presumably due to the formation of additional isolated hydroxyl groups, e.g., after extraction of Ti atoms together with next-neighbor SiO_4_ tetrahedra [26]. This is also consistent with the observed decrease of this peak after calcination due to the expected surface dehydroxylation resulting from the high-temperature treatment. The obtained surface basic site density and corresponding intrinsic adsorption capacity are presented in Table 2.

### Surface characterization of the ETS-10-based titanosilicates by ^29^Si MAS NMR

**Figure 13:**
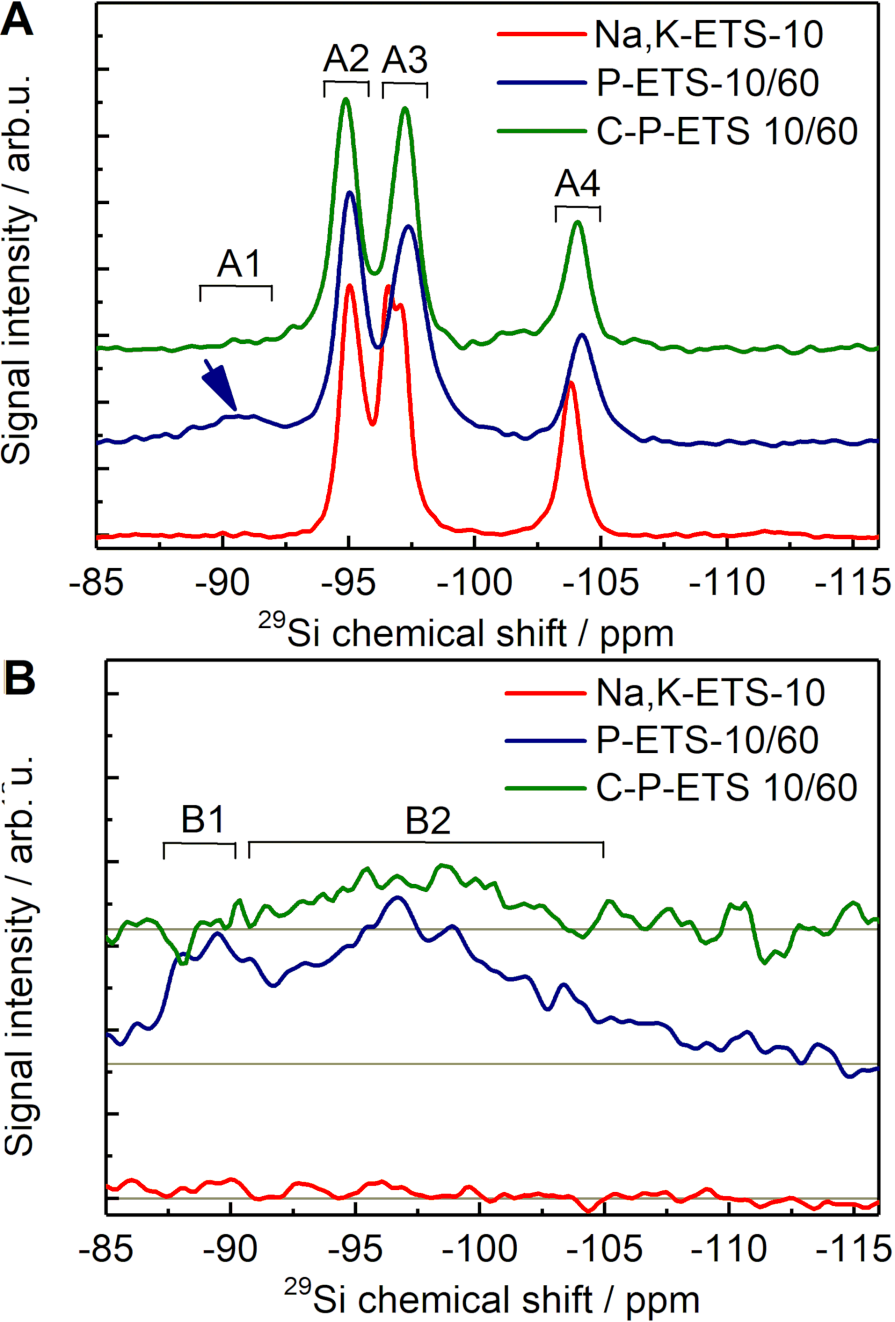
The HPDEC (A) and CP (B) ^29^Si MAS NMR spectra obtained for Na,K-ETS-10 (red), P-ETS-10/60 (blue) and C-P-ETS 10/60 (green) at 12 kHz and 298 K. The arrow in (A) indicates a detectable signal centered at 90 ppm.

For quantitative analysis of the surface groups present in the titanosilicates before and after treatment, ^29^Si MAS NMR was used. Figure 13A demonstrates the measured high-power decoupled (HPDEC) spectra of the ETS-10-based samples. The spectrum of the Na,K-ETS-10 exhibits three distinguishable peaks at −95.1 ppm (A2), −96.8 ppm (A3) and at −103.8 ppm (A4) with calculated relative peak areas after deconvolution of 40, 40 and 20%, respectively (see Table 3). This is consistent with the structural data provided for ETS-10 in [23]. The two resonances at −95.1 ppm and −96.8 ppm are assigned to the Si bonded via O atoms to three Si atoms and one Ti atom, i.e., Si(3Si, 1Ti). The third peak at −103.8 ppm originates from the Si coordinated through O atoms to other four Si atoms, i.e., Si(4Si, 0Ti). Due to the very low number of protons in the Na,K-ETS-10 sample, the signal is not observable in the respective cross-polarization (CP) spectrum presented in Figure 13B. It is worth mentioning that the CP technique facilitates polarization transfer from protons to silicon to acquire spectra of the latter.

**Table 3:** Assignment and estimated relative peak areas of the ^29^Si MAS NMR peaks presented in Figure 13A.

Titanosilicate	Chemical shift / ppm	Peak labelling	Si coordination	Relative peak area^a^

Na,K-ETS-10	−90.0	A1	Si (2Si, 1Ti, 1OH)	0.002
	−95.0	A2	Si (3Si, 1Ti)	0.400
	−97.1	A3	Si (3Si, 1Ti)	0.401
	−103.9	A4	Si (4Si, 0Ti)	0.197
P-ETS-10/60	−90.0	A1	Si (2Si, 1Ti, 1OH)	0.058
	−95.0	A2	Si (3Si, 1Ti)	0.430
	−97.3	A3	Si (3Si, 1Ti)	0.339
	−104.2	A4	Si (4Si, 0Ti)	0.172
C-P-ETS-10/60	−90.0	A1	Si (2Si, 1Ti, 1OH)	0.023
	−95.0	A2	Si (3Si, 1Ti)	0.486
	−97.3	A3	Si (3Si, 1Ti)	0.307
	−104.2	A4	Si (4Si, 0Ti)	0.184

^a^Calculated after peak deconvolution.

The HPDEC spectrum of the P-ETS-10/60 sample in Figure 13A shows four peaks in the range between −85 and −110 ppm, where the tetracoordinated Si might occur [49]. The presence of an additional peak at −90 ppm (A1) and a relative area of 6% was assigned to Si (2Si, 1Ti, 1OH) resulting from the intense surface hydroxylation after treatment of the titanosilicate with H_2_O_2_. This hypothesis was confirmed by efficient cross-polarization from the protons of the OH groups, accompanied by a significant enhancement of the signal at 90.0 ppm (B1) in Figure 13B. The rest of the signals in the CP spectrum of P-ETS-10/60 (B2) originate from the Si(3Si, 1Ti). The peaks at −95.0 ppm and −97.4 ppm of the HPDEC spectrum are similar to those seen for Na,K-ETS-10, i.e., coordinated as Si(3Si, 1Ti). They show relative areas of 43% and 34%, respectively. In comparison to the spectrum of Na,K-ETS-10, the intensity of the peak centered at −97.3 ppm is reduced due to the partial hydroxylation of the silicon atoms after treatment. In the CP spectrum, the hydroxylated species of Si(3Si, 0Ti, 1OH) exhibit a broad peak at −96.4 ppm, while in the HPDEC spectrum they are not visible, probably due to the low intensity and the resonance overlap with the highly abundant Si(3Si, 1Ti) species. The peak at −104.2 ppm is assigned to Si(4Si, 0Ti) and has a slightly reduced relative area of 17%. The loss in silicon atoms with this resonance comes mainly from the hydroxylation that shifts the peak of the resulting species to −96.4 ppm.

The HPDEC spectrum of the C-P-ETS-10/60 material in Figure 13A clearly shows that the Si(2Si, 1Ti, 1OH) species at −90.0 ppm have vanished. In the CP spectrum in Figure 13B, this is confirmed by the absence of the respective peak at −90 ppm. Within the detection limit of the experiments, this suggests that the OH groups have been removed during the calcination for the case of Si(2Si, 1Ti, 1OH). At the same time, the signal at −95.0 ppm reached an area of 49%. This increase after calcination presumably comes from the formation of Si(3Si, 1Ti) at a chemical shift of −95.0 ppm from the available number of Si(2Si, 1Ti, 1OH) species. The area of the peak at −96.2 ppm is lower compared to the same one measured for P-ETS-10/60 due to smaller overlapping signal of Si(3Si, 0Ti, 1OH) seen after calcination. In the CP spectrum, the impact of the polarization transfer to the species of Si(3Si, 0Ti, 1OH) from the OH groups is still seen, leading to a broad peak at −95.5 ppm with lower intensity than prior to calcination. This suggests that dehydroxylation of Si with adjacent Ti is more effective than for the species surrounded by four Si atoms. In the HPDEC spectrum, the peak at −104.2 ppm, which is assigned to Si(4Si, 0Ti), has a slightly higher relative area of 18% after calcination. This increase might come from the additional formation of Si(4Si, 0Ti) from the previously hydroxylated species after calcination. For convenience, the assigned elements are illustrated in Figure S13 (A-C) of Supporting Information File 1.

### The state of Ti sites probed by EPR spectroscopy

**Figure 14:**
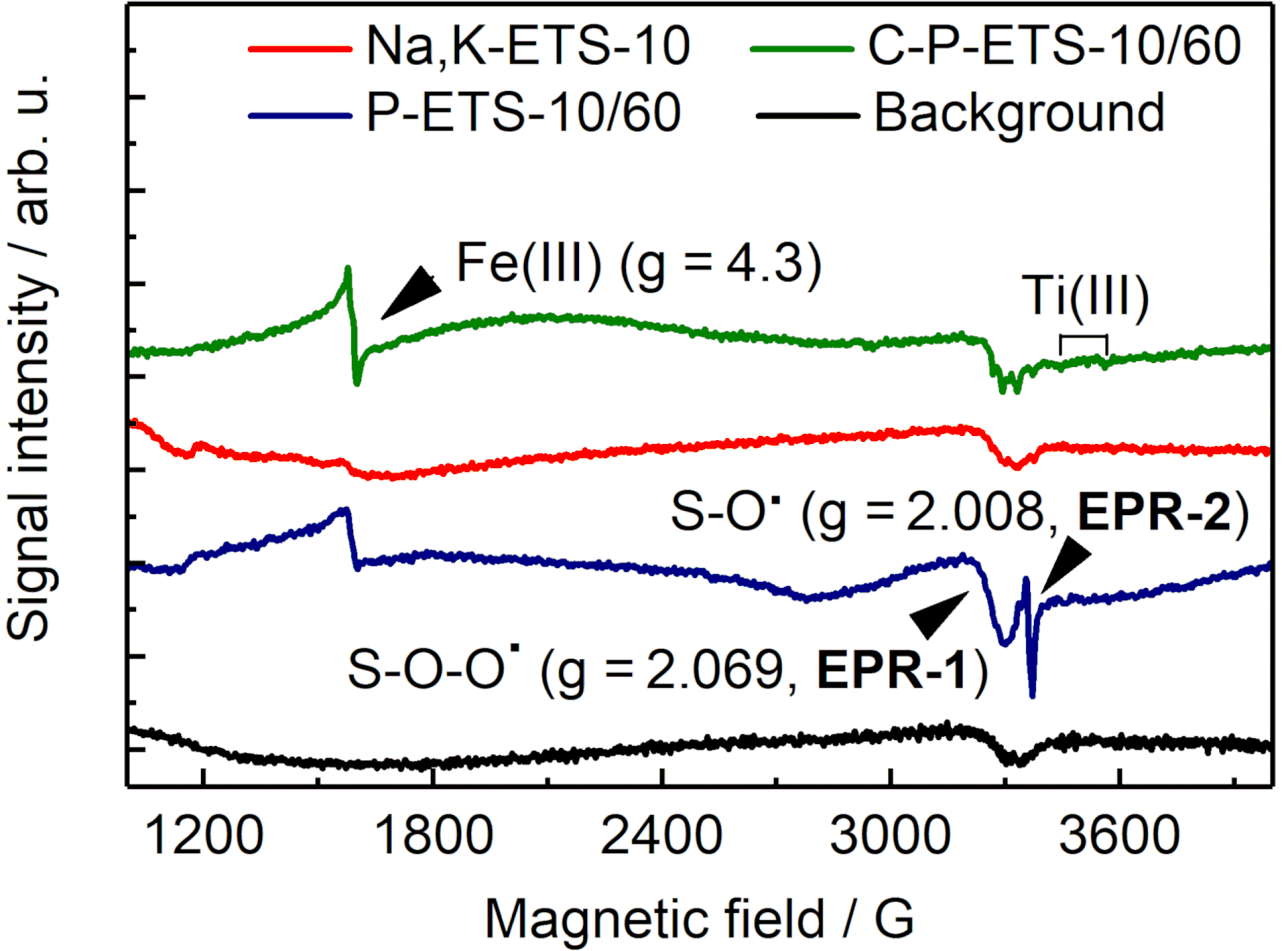
The EPR spectra measured with Na,K-ETS-10 (red), P-ETS-10/60 (blue) and C-P-ETS-10/60 (green) at 70 K. The background spectrum taken on an empty sample tube is presented for comparison (black).

It has been demonstrated that application of post-synthetic treatment to titanosilicates can change the state of titanium atoms from Ti(IV) to catalytically active Ti(III) [50]. Such a reduction to paramagnetic Ti in ETS-10 titanosilicates can occur as a result of high-temperature treatment under vacuum [51], treatment with H_2_ or CO at moderate to high temperatures [52], ion exchange [53–54], UV [55] or γ-irradiation [51]. To characterize the state of Ti in the titanosilicates participating in catalytic tests and the possible presence of impurities, the EPR spectra of these samples were measured at 70 K (Figure 14).

The spectrum of the parent K,Na-ETS-10 expectedly showed no signals of Ti(III) around 3500 G due to its oxidation state Ti(IV), which is diamagnetic and thus EPR-silent. Both samples, C-P-ETS-10/60 and P-ETS-10/60, display a typical signal of high spin Fe(III) of dispersed paramagnetic centers. Their *g*-value of 4.3 is indicative of a large axial zero-field splitting (ZFS) parameter (*D* >> 10 GHz) and a ratio between rhombic and axial ZFS of 1/3. Such Fe(III) centers have been commonly observed in zeolite and silica materials and were assigned to iron sites with a distorted tetrahedral coordination geometry [56–57]. In addition, P-ETS-10/60 exhibits signals at *g* = 2.069 and *g* = 2.008, which can be assigned to peroxy radicals Si–O–Ο^•^ (peak EPR-1) and non-bridging oxygen Si–O^•^ (peak EPR-2) resulting from the desilication process [58], respectively. It is worth noting that no EPR signals from Ti(III) species with typical *g* values in the range of 1.89–1.97 were observed for all investigated materials. This suggests that the presence of the paramagnetic Ti(III) in the treated titanosilicates is negligibly small.

### Variable temperature and 2D-exchange hyperpolarized ^129^Xe NMR experiments for probing the interconnectivity between micro-, meso- and macropores in titanosilicates

The introduction of mesopores after the post-synthetic treatment into initially microporous material does not necessarily lead to the organization of micro- and mesopores in a hierarchical manner. A prominent example is the dealumination of the USY zeolite by steaming as reported in [59], which leads to the appearance of separated domains of micropores and mesopores as large as several micrometers. In such a case, it is expected that a significant portion of the mesopores might be not accessible for bulky reactants, such as triolein, being too large to diffuse through the micropores. In the case of treatment of ETS-10 titanosilicates with H_2_O_2_ at elevated temperatures, if accompanied by microexplosions [39], the appearance of such domains is possible. To address this, a number of NMR experiments with adsorbed Xe possessing a polarizability that is high enough to distinguish between micro-, meso-, and macropores were conducted [60]. The respective signals of Xe differing by the chemical shift, under the assumption of a slow or moderate chemical exchange between different types of pores, would deliver quantitative information on their interconnectivity [61–62].

**Figure 15:**
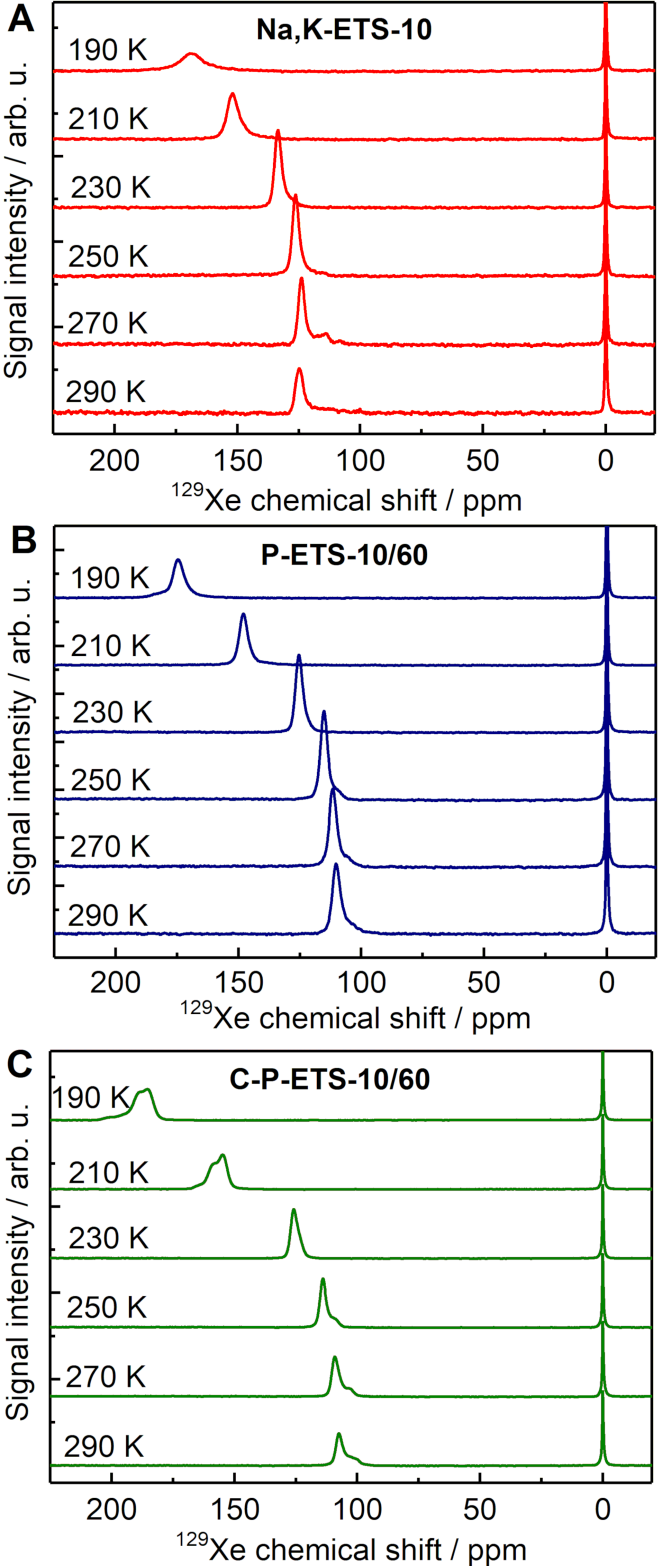
Variable temperature HP ^129^Xe NMR spectra acquired on Na,K-ETS-10 (A, red), P-ETS-10/60 (B, blue) and C-P-ETS-10/60 (C, green). The chemical shift scale is referenced to the signal of free Xe gas measured in an empty tube at 290 K.

Figure 15 represents the spectra from variable temperature experiments using the hyperpolarized ^129^Xe gas. The signals at 0 ppm are assigned to free Xe gas in the detectable part of the space above the sample compartment and to Xe located between the crystals. Other signals at higher ppm values are assigned to Xe adsorbed inside pores of titanosilicates. In the case of Na,K-ETS-10 (Figure 15A), the chemical shift of adsorbed Xe shifts monotonically from 125 ppm at 290 K to 169 ppm at 190 K due to growing Xe density with decreasing temperature. The peak is assigned to Xe adsorbed in the 12-member rings, while the 7-member ring, according to [63], is not accessible. For P-ETS-10/60, for all temperature ranges studied, one peak with a chemical shift extending from 110 ppm to 174 ppm is observed (Figure 15B). If the presence of mesopores can be confirmed by N_2_ sorption and Hg porosimetry, this result might originate solely from the rapid Xe exchange between micro- and mesopores highly interconnected to each other. An alternative explanation that the appearance of mesopores with multiple surface hydroxyl groups are not accessible for Xe due to too low hydrophobicity seems to be improbable. A similar observation is seen for C-P-ETS-10/60 with a Xe chemical shift variation from 108 ppm to 185 ppm, as presented in Figure 15C. However, another signal with a very close chemical shift value can be identified. This signal might originate from Xe penetration into the 7-member ring pores, and the amount of present OH groups of which might decrease after calcination, thus enhancing their accessibility. Earlier, it was demonstrated that Xe can squeeze into the pores with dimension as small as 0.37 nm [64]. Two-dimensional exchange spectroscopy (EXSY) experiments were conducted to probe the exchange between the micro-/mesopores and of the space between the crystals. The aim here was to investigate the possible presence of barriers on diffusion for molecules entering or exiting the pore space of the titanosilicate crystals [65]. All studied titanosilicates demonstrated detectable cross-peaks for 100 ms mixing time originating from the entering and exiting Xe, suggesting sufficient exchange on this time scale (see Figures S12 of Supporting Information File 1).

### Probing the mesopore accessibility for triolein using PFG NMR with ultrahigh gradients

Post-synthetic modification has resulted in the appearance of a fraction of mesopores ranging from a few to a few tens of nanometers (see, e.g., Figure 9B). This size is large enough to accommodate the long-chain triolein or methyl oleate with a largest dimension of ≈2.5 nm of a single aliphatic part and a certain degree of chain flexibility [66]. However, the data of N_2_ sorption or CO_2_-/NH_3_-TPD do not guarantee that the mesopores will become accessible for these molecules in a reaction. In our recent study [67], we demonstrated the applicability of pulsed-field gradient (PFG) NMR spectroscopy for measuring self-diffusion coefficients of the long-chain hydrocarbons (up to C19) confined to the nanopores of a catalyst. Here, we attempted to use this approach for clarification of the mesopore accessibility by the direct diffusion measurement using triolein. Due to the unique design of the probe (combination of the ultrahigh field gradients with the possibility of temperature control up to 463 K), it became possible to probe diffusion of nanoscopically confined triolein (C_57_H_104_O_6_) at 403 K resembling temperature used during the catalytic tests.

**Figure 16:**
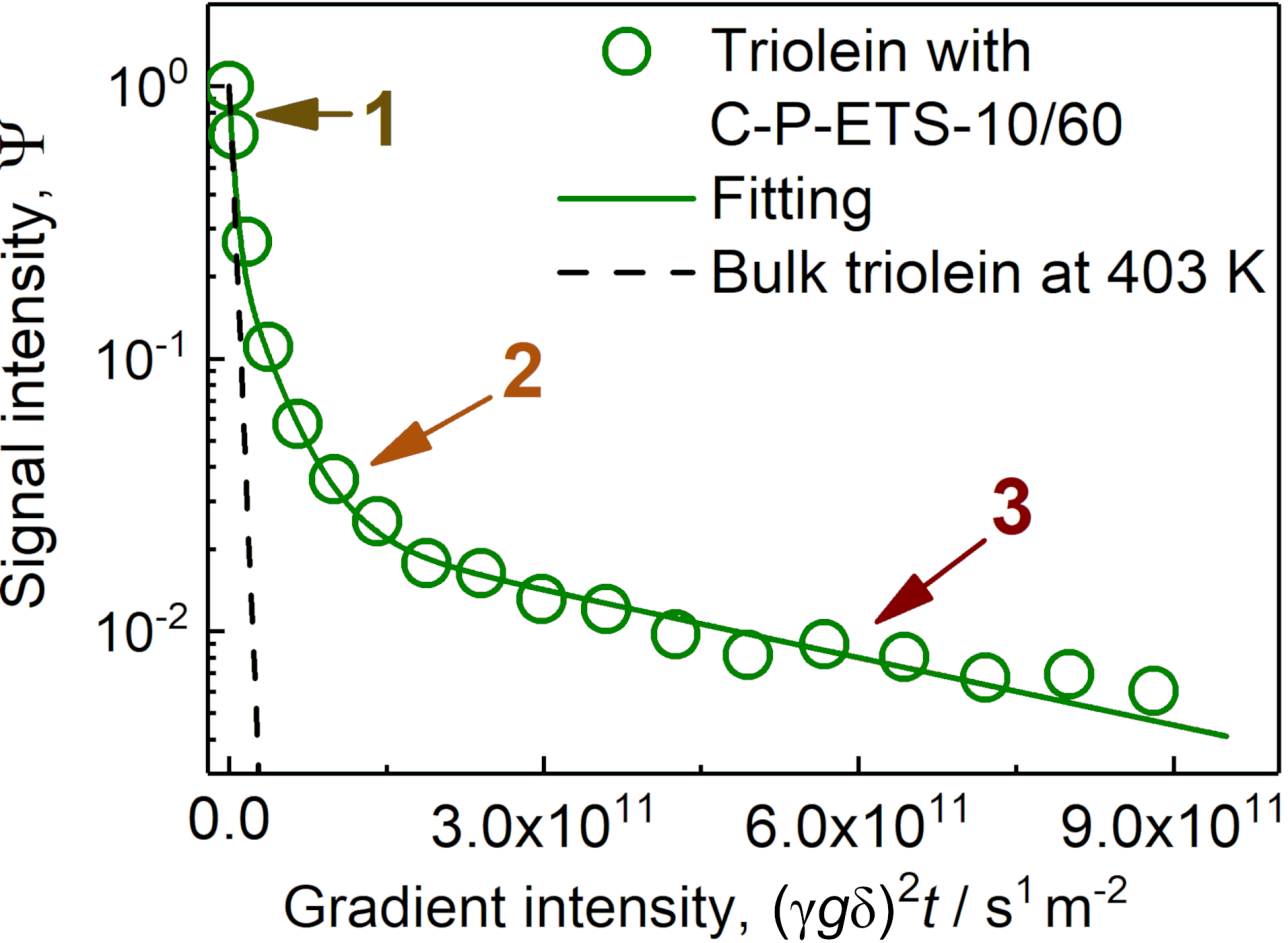
^1^H PFG NMR diffusion attenuation curves of bulk triolein (dashed line) and triolein in oversaturated C-P-ETS-10/60 titanosilicate at 403 K. The solid line represents converged fitting using Equation 2 with i = 3. The ranges where the corresponding diffusion mode dominates are shown by the arrows.

**Figure 17:**
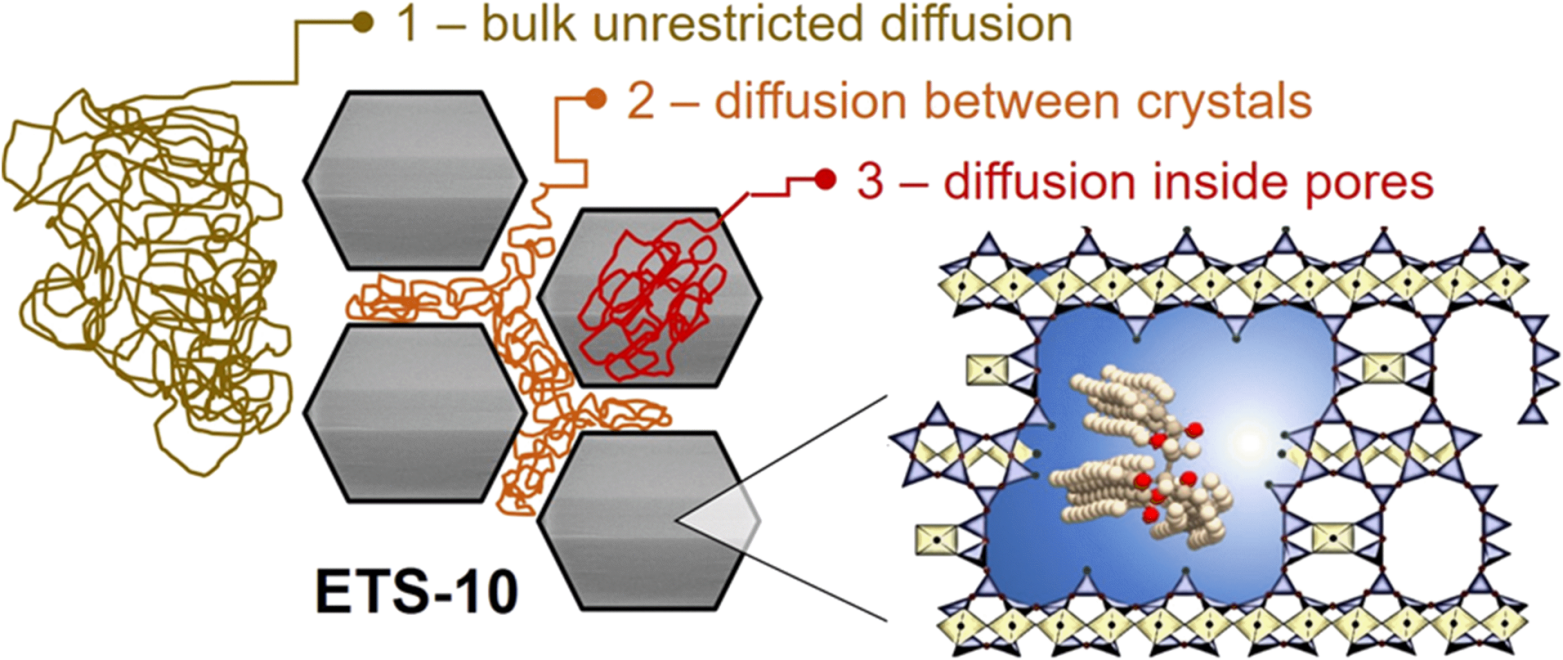
Schematic representation of the diffusion modes denoted as 1, 2 and 3 in the Figure 16 as observed by PFG NMR. The inset represents part of the treated ETS-10 framework with triolein inside it. The sketched trajectories are not to scale – see Table 4 for measured RMSD values.

Figure 16 demonstrates the measured diffusion attenuation curve for triolein in C-P-ETS-10/60, which could be satisfactorily fitted using Equation 2 and a sum of three diffusion modes. The result of the fitting procedure is presented in Table 4. In the utilized oversaturated sample, that is, when the bulk excess was present, one component (the fastest amongst all others and labelled as “1”) shall be assigned to the diffusion of the bulk triolein. This value was also measured in a separate experiment with triolein only, resulting in 2.0 × 10^−10^ m^2^ s^−1^ (see the dashed line in Figure 16) and was fixed during the fitting procedure. The second mode “2” (with diffusivity 2.9 × 10^−11^ m^2^ s^−1^) presumably originates from the diffusion between the ETS-10 crystals and the film diffusion on the surface of the crystals. It is approximately one order of magnitude lower than the bulk diffusivity. The mode revealing the slowest diffusion (1.9 × 10^−12^ m^2^ s^−1^) was assigned to triolein located within the mesopores of ETS-10 crystals. The root mean squared displacement (RMSD) estimated using the Einstein relation resulted in 0.5 μm, which is much smaller than the average crystal size measured by laser diffraction (≈30 μm). This suggests that the mode “3” represents intrinsic intracrystalline diffusivity with a negligibly small influence of the possible diffusion exchange with the intercrystalline space during the used diffusion time (20 ms). It is worth mentioning that in both catalysts, microporous Na,K-ETS-10 and micro–mesoporous P-ETS-10/60, the amount of triolein was below the detection limit. While for the microporous titanosilicate it was expected that the too small pores prohibit accommodation of triolein molecules. For P-ETS-10/60 this might result from the decreased surface hydrophobicity (as seen by the ^29^Si NMR and indirectly by the NH_3_-TPD), preventing efficient diffusion into the mesopores of the crystals. The discussed scenarios are illustrated in Figure 17 with representation of the respective diffusion modes. To the best of our knowledge, this study represents the first direct measurement of the self-diffusion coefficient of (any) triglyceride or oil (as their mixture) confined to nanopores of (any) catalyst.

**Table 4:** Result of the fitting of the data presented in Figure 16: the self-diffusion coefficients and the resulting root mean squared displacements (RMSDs).

Diffusion mode	Self-diffusion coefficient / m^2^ s^−1^	RMSD / μm

1 (fast)	2.0 × 10^−10^	4.91
2 (intermediate)	(2.9 ± 0.2) × 10^−11^	1.9 ± 0.1^a^
3 (slow)	(1.9 ± 0.3) × 10^−12^	0.5 ± 0.1^a^

^a^The upper uncertainty limit of the RMSD is taken to be equal to the lower one, which is larger.

Based on the results of NH_3_-TPD, CO_2_-TPD, ^29^Si MAS NMR, EPR and PFG NMR, the structures of the titanosilicates with summarized significant elements (e.g., acid and basic sites as seen by TPD, structural defects caused by post-synthetic treatment, presence of hydroxyl groups, pore accessibility for triolein) are presented in Figure S13 of Supporting Information File 1.

### Transesterification of triolein to methyl oleate over the CaO- and ETS-10-based catalysts

**Figure 18:**
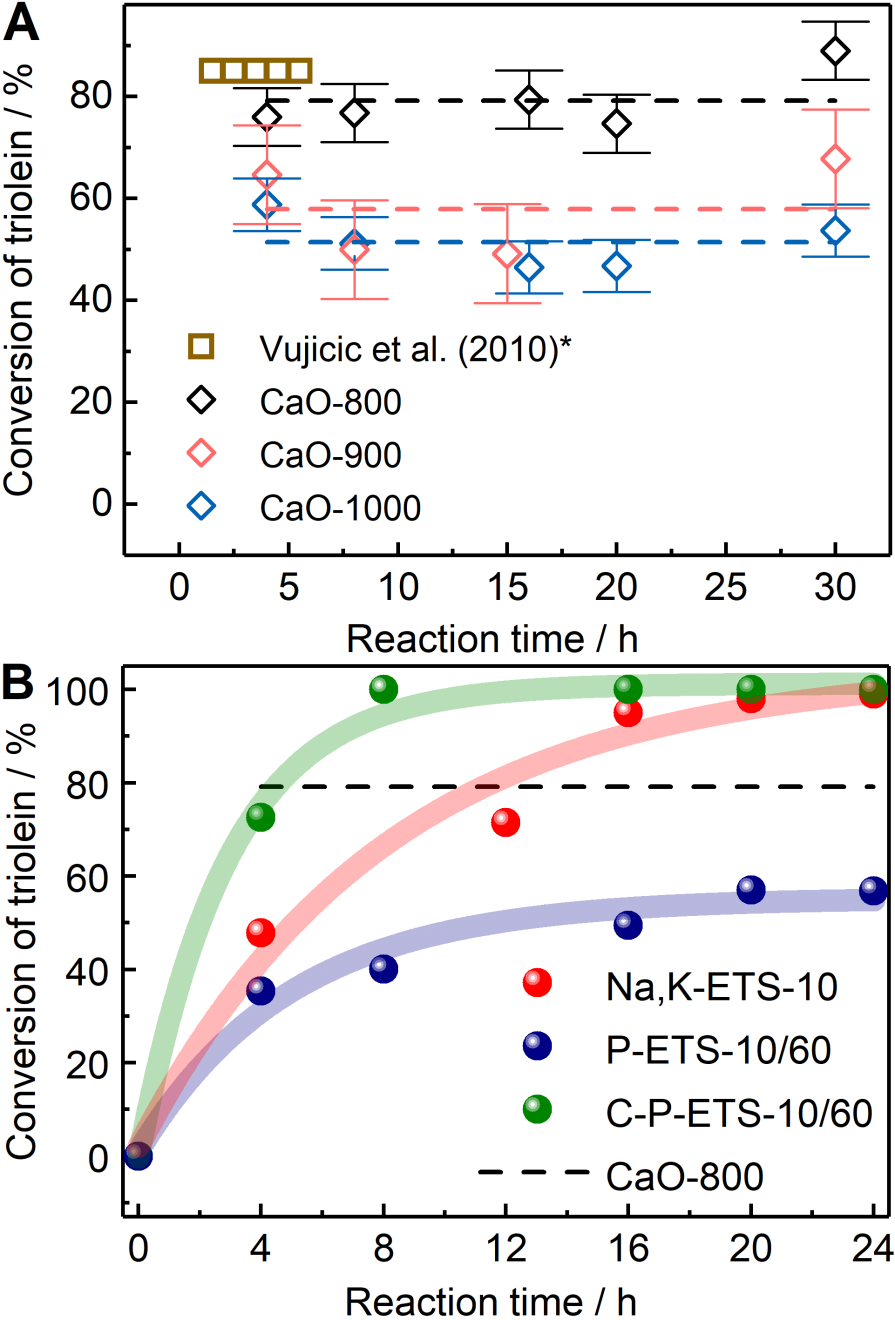
Conversion of triolein over CaO- (A) and ETS-10-based (B) catalysts at 403 K measured for different reaction times. The dashed lines in A represents an average conversion for each catalyst calculated from the values obtained for different times assuming no time dependence. For comparison, the data obtained on the transesterification of sunflower oil (*recalculated for conversion of oil) at 393 K over CaO from [74] are presented. The dashed line in B was adapted from A and represents the conversion over the most active CaO catalyst (CaO-800). Semitransparent lines and conversions in B at time 0 h are used to guide the eye.

Prior to catalytic experiments with titanosilicates, the conversion of triolein was tested over CaO as the reference and well-studied for transesterification of various triglycerides with methanol, mostly for biodiesel production [68–74]. Additionally, the goal was to have the identical reaction conditions in experiments with titanosilicates for the possibility of direct comparison. Depending on the activation (calcination) conditions, the activity of the CaO-based catalysts was observed to depend strongly on the calcination temperature. In [74], an increase in the yield of FAME from 17 to 85% after 5.5 h of reaction time was observed upon the change of the calcination temperature from 773 to 1173 K. This trend was observed in multiple reports in which CaO was used [73]. It is associated with the minimization of Ca(OH)_2_ and CaCO_3_ during calcination, which have lower activity compared to CaO. However, after a certain calcination temperature corresponding to the highest possible activity, a decrease in the activity for the catalyst calcined at even higher temperatures was reported [70–71]. This decrease is attributed to the loss of accessible surface area resulting from the partial sintering of CaO crystals at high calcination temperatures (usually above 1100 K). A similar observation was found for catalysts prepared and used in the present study. In Figure 18A, the catalyst CaO-800 calcined at 1073 K exhibited the highest conversion of triolein amongst those tested, that is, 79% after 4 h of reaction. This value is consistent with the reported FAME yields in the transesterification of sunflower oil with methanol at 393 K over the catalyst identically prepared and calcined at 1173 K (presented by squares for comparison). The CaO-900 and Ca-1000 reached 57% and 51% of conversion after 4 h, respectively. The observed lower conversion with increasing catalyst calcination temperature is consistent with the SEM and N_2_ sorption results, demonstrating a decrease in the specific surface area (see Table S4 of Supporting Information File 1).

No further change in conversion was observed for reaction times higher than 4 h. The transesterification is a reversible process with possible back reactions to form mono-, di-, or triglycerides. In [75], it was demonstrated that the biodiesel yield over the CaO catalyst can be improved by introducing excess amounts of methanol to force the reaction towards the formation of biodiesel, that is, by increasing the methanol-to-triglyceride molar ratio. When this ratio reached 9:1, the yield reached the maximum value with no further increase. In the present study, a 36:1 ratio was selected to assure negligible impact of the back reaction on the measured triolein conversions. Thus, the observed catalyst deactivation was attributed to the problem of catalyst leaching during the course of the reaction [76].

The results of the catalytic studies with the prepared titanosilicates are presented in Figure 18B. The parent Na,K-ETS-10 showed 48% of conversion after 4 h of reaction. The conversion continued to increase, reaching 100% after 20 h. This activity seems to be slightly lower than those reported in the literature, in which ETS-10 catalysts were used in transesterification reactions with various triglycerides at comparable temperatures [20,77–78]. For example, in [20], 80% of the conversion has been achieved after 2 h at 393 K. It should be mentioned, however, that it was achieved under the permanent stirring conditions and with the catalyst from the former Engelhard Corporation (Iselin, NJ) having an average crystal size ranging from a few hundred nanometers [50] to a few micrometers [79]. In the conducted catalytic tests, the tube was shaken only once and left undisturbed for the entire reaction time. In addition, the average crystal size of the prepared Na,K-ETS-10 was 30 μm, reducing the accessible surface area outside the crystal per mass of a catalyst compared to the commercial ones.

The introduction of mesopores by treatment with H_2_O_2_ aimed at increasing the total accessible surface area of the catalysts; for P-ETS-10, it led to the lower activity compared to the microporous Na,K-ETS-10. The maximum conversion of 57% was reached only after 20 h of reaction. This observation, being counterintuitive at a first glance, can be explained by the reduced hydrophobicity of the surface after treatment, also on the outer crystal surface. This is consistent with the absence of detectable adsorbed triolein inside the pores of P-ETS-10. A similar effect has been observed in [80] for the methyl oleate epoxidation with H_2_O_2_ over the parent (microporous) and alkaline treated (hierarchical) titanium silicalite-1. Despite the mesopores present in the treated sample, the conversion of methyl oleate decreased by ≈50%, which has been attributed to the decreased hydrophobicity after treatment. To address this effect directly, further studies including the measurement of water sorption isotherms to obtain the “hydrophilicity index” according to Thommes et al. [81] are planned.

In the case of the calcined hierarchical titanosilicate C-P-ETS-10, a dramatic increase in activity is observed, as compared to Na,K-ETS-10 and P-ETS-10/60, showing full conversion already after 8 h. After 4 h, the conversion of triolein reaches ≈73%, becoming comparable to that of CaO-800 (≈76%). The latter remains unchanged over the time, while the C-P-ETS-10 exhibits a further increase up to 100%. The observation of such activity appeared despite the lowest surface basic site density and intrinsic base adsorption capacity amongst the tested titanosilicates measured by CO_2_-TPD. It is worth mentioning that CO_2_-TPD does not distinguish between the surface of micro- and mesopores, while the active sites (the Brønsted strong basic sites) located only in mesopores can be accessible during the reaction. Thus, the “effective” basicity seen by the reactants might differ from the measured values. In addition to this, as is evident from the results of DTA and ^29^Si NMR, calcination leads to the partial surface dehydroxylation. This might improve the accessibility of mesopores for triolein via the increased surface hydrophobicity. Additionally, the hydrophobicity of the outer crystal surface is expected to be restored after calcination, adding to the resulting activity of the C-P-ETS-10/60 catalyst.

Using the obtained self-diffusion coefficient of pure triolein inside the mesopores of C-P-ETS-10 at 403 K, a simple estimate results in ≈150 s for the upper time limit needed to cross a crystal with 30 μm size. This value, which under the reaction conditions might become lower due to the presence of methyl oleate and methanol, is much smaller than the time needed to reach full conversion (8 h). As it has been reported in [82], for the hydrogenation of 1,3,5-triisopropylbenzene with a critical diameter of ≈0.95 nm over the Pt supported on controlled pore glass with 80 nm pore width and particle size of 50–100 μm at 373 K, a clear indication of diffusion limitation was observed. Thus, with the larger size of triolein and the smaller average mesopore size (≈30 nm) of C-P-ETS-10/60, it can be expected that the reaction will be diffusion limited under the applied conditions. However, in order to properly address this question, further studies with a variation of the particle sizes and more frequent sampling for obtaining the initial reaction rates are needed.

## Conclusion

The applied hydrothermal synthesis of the microporous ETS-10 titanosilicate (Na,K-ETS-10) resulted in crystals with an average size of ≈30 μm without structural defects visible by SEM and TEM, but with the presence of a small fraction (less than 5%) of alternative phases (such as ETS-4, AM-1, or quartz) appearing as side products. This was confirmed by X-ray diffractograms and TEM, revealing the high crystallinity of the material.

Post-synthetic treatment with H_2_O_2_ at 423 K led to the successful introduction of mesopores in the range of 4–40 nm interconnected with the micropores in a hierarchical manner. The latter has been confirmed in the hyperpolarized ^129^Xe NMR experiments by monitoring the fast Xe exchange between micro- and mesopores on a timescale of microseconds. Along with this, such a treatment is accompanied by the appearance of additional OH groups on the surface of the created mesopores originating from the partial removal of TiO_6_ octahedra and SiO_4_ tetrahedra from the framework and subsequent protonation of the oxygen radicals. The quantitative information of the surface modification was obtained from the ^29^Si MAS NMR experiments. This step led to a 2.6-fold increase of the intrinsic acid adsorption capacity of the treated catalyst (P-ETS-10/60) compared to the Na,K-ETS-10 as probed by NH_3_-TPD, presumably due to intense surface hydroxylation, and therefore, reduction in hydrophobicity. The latter has led to an even lower activity compared to the parent titanosilicate. The basicity probed by CO_2_-TPD exhibited two peaks for Na,K-ETS-10 that were assigned to the Na,K-cations as weak Lewis bases in the low-temperature region and to the strong Brønsted basic oxygen atoms coordinated as Si–O–Ti in the framework. The application of the H_2_O_2_ treatment only slightly affected the intrinsic base adsorption capacity, while shifting the CO_2_ desorption to temperatures above 600 K.

The subsequent calcination of the H_2_O_2_ treated titanosilicate has led to notable surface dehydroxylation while preserving mechanical stability, high material crystallinity and a hierarchical pore network. The surface basicity after calcination as probed by CO_2_-TPD was found to be the lowest amongst other titanosilicates. Despite this, the activity of the catalyst in the triolein transesterification was observed to be higher than that of the Na,K-ETS-10 and P-ETS-10/60. This result was assigned to the drastically improved accessibility of the active sites on the surface of the mesopores for reactants, which was also confirmed by the direct diffusion measurements using PFG NMR with ultrahigh gradients. Further comparison of the triolein conversion with CaO-based catalysts tested under the same conditions revealed comparable values after 4 h of the reaction course, i.e., 73% and 76% for the calcined hierarchical ETS-10 and CaO catalysts, respectively. Remarkably, while the conversion over the latter did not further change due to stability issues, an ETS-10-based catalyst revealed achievement of 100% already after 8 h. This observation suggests that the ETS-10 titanosilicates, when appropriately treated, exhibit comparable behavior to the CaO catalysts activity in the heterogeneously catalyzed transesterification of triglycerides, but may outperform them in terms of stability and ability for regeneration.

## Experimental

### Materials and chemicals used in the preparation of CaO- and ETS-10-based catalysts

Calcium oxide of technical grade (Centrochem Co., Serbia) was used as a precursor in the preparation of CaO-based catalysts. For the preparation of ETS-10, sodium silicate (Na_2_SiO_3_, 34.5–36.0 wt % SiO_2_, 17–19 wt % Na_2_O, donated by PQ corporation), titanium isopropoxide (TIP, 97 wt %, Sigma-Aldrich), hydrochloric acid (HCl, 35–37 wt %, VWR Chemicals), sodium chloride (high-purity grade, VWR Chemicals), and potassium fluoride (KF, ≥99 wt %, Fluka, Sigma-Aldrich) were used. In the post-synthetic treatment of ETS-10, hydrogen peroxide (H_2_O_2_, 30 wt %, Merck Millipore) and distilled deionized water (DDW, 0.055 μS conductivity, PureLab flex 3 & 4 water purification systems) were used.

### Chemicals used in the transesterification reaction and gas chromatography

Triolein (>99 wt %, Sigma-Aldrich) and methanol (99 wt %, VWR corporation) were used in the transesterification reaction. Methyl oleate (>99 wt %, Sigma-Aldrich), 1,3-diolein (>99 wt %, Sigma-Aldrich), 1-oleoyl-*rac*-glycerol (≥99 wt % monolein, Sigma-Aldrich), and glycerol (99 wt %, Sigma-Aldrich) were used for calibration of analytes. All mentioned materials and chemicals were used without further purification.

### Preparation of the CaO-based catalysts

The CaO precursors were activated at temperatures of 1073 K, 1173 K, and 1273 K for 3 h under static air conditions in the Elektron Co. furnace. After calcination, the catalysts were kept in a desiccator. The obtained catalysts were denoted according to the applied calcination temperatures as CaO-800, CaO-900, and CaO-1000, respectively.

The CaO-based catalysts were then shaped into pellets by using a hydraulic press (Womax Co.) at a pressure of 5 t. Afterwards, the pellets were ground and sieved in order to obtain fractions between 1.0 and 1.5 μm.

### Preparation of ETS-10-based catalysts

ETS-10 molecular sieves were prepared by hydrothermal synthesis following a reported procedure [32]. Initially, a visually transparent silica-containing solution was prepared by dilution of the 9.8 mL aqueous solution of 51–55 wt % Na_2_SiO_3_ with 45.2 mL of DDW in a 250 mL flask. An aqueous solution of NaCl (3.4 g of NaCl dissolved in 10.0 mL of DDW) was subsequently added into the flask with the silica-containing solution in it. A titania-containing solution was prepared by dissolving 5.4 mL TIP and 3.0 mL HCl in 12.6 mL DDW in a 100 mL flask. Both the silica- and titania-containing solutions were prepared under vigorous stirring for 2 h at room temperature.

The prepared titania-containing solution was then added dropwise using a pipette into the silica-containing solution while stirring, resulting in the appearance of an opaque mixture. After this, the mixture was stirred for 1 h at room temperature. A KF-solution was prepared by dissolution of 0.1 g of KF in 2.5 mL of DDW, resulting in a 0.0011 g mL^−1^ concentration. The prepared amount of a KF solution was further added into 87.5 mL of the titania/silica-containing solution. The resulting molar composition of the obtained 90 mL solution is the following SiO_2_/TiO_2_/HCl/Na_2_O/NaCl/KF/H_2_O = 5.56:1:1.94:2.64:3.22:0.09:216.

The solution was then further aged for 16 h in a flask at room temperature for polymerization to obtain –[–Ti–O–Ti–]–, –[–Si–O–Si–]–, and –[–Ti–O–Si–]– containing seeds. After aging, the 90 mL solution was divided into two 45 mL parts, each of which was placed into the 70 mL polytetrafluoroethylene (PTFE) reactor. The reactor was closed with a PTFE cap and inserted into a homemade stainless steel autoclave. The design of the autoclave is similar to a standard acid digestion vessel. The autoclave was then closed with a stainless steel screw-tightened cap. Both autoclaves were placed simultaneously into a preheated (473 K) VWR dry air convection oven and kept inside for 96 h at this temperature. Afterwards, the autoclaves were removed from the oven and quenched in cold water down to room temperature. A white powder was observed at the bottom of the reactor. The solutions from the reactor containers were transferred to 50 mL centrifuge tubes for further centrifugation for 10 min at 3000 rpm in a Thermoscientifc Heraeus Megafuge 8 centrifuge. Three washing steps with DDW and a subsequent centrifugation step were taken for the removal of residual components remaining after synthesis. In a final step, the powder was dried in a convection oven at 423 K for 24 h. Then, the pre-dried powder was placed into the vacuum oven and kept inside at 1 kPa pressure and 373 K for 24 h to remove residual moisture, which might be present inside the pores. The obtained titanosilicate was denoted as Na,K-ETS-10.

A graphical sketch of the synthesis procedure and calculations of the mixture volumes are provided in section S1 of Supporting Information File 1.

### Variation of KF- and HCl during synthesis

The ETS-4 phases were minimized in the as-synthesized ETS-10 by varying the HCl concentration from 0.033, 0.044, 0.047 to 0.05 g mL^−1^ (3, 4, 4.2, 4.6 mL) in the solution (see section S6 of Supporting Information File 1). This implies that four 90 mL solutions were prepared with four different HCl concentrations. The procedure followed was reported by Lv et al. [29]. The addition of a HCl step was carried out similar as mentioned in the section before. After preparing the 90 mL solution, the pH value was determined using a Mettler-Toledo Seven Go DuoPro with a Mettler Toledo InLab Expert Pro ISM IP67 sensor probe. The remaining steps for the preparation were similar as mentioned in the section before.

Another investigation was also carried out to minimize AM-1 along with ETS-4 in the Na,K-ETS-10 (see section S7 of Supporting Information File 1). The crystallization of different titanosilicate phases were observed by varying the concentration of KF and HCl in the different synthesis solutions. The concentration for HCl was varied from 0.026 g mL^−1^ to 0.068 g mL^−1^ in the 90 mL solution while keeping the KF concentration constant. Similarly, the concentration of KF was varied from 0.007 g mL^−1^ to 0.026 g mL^−1^ in the 90 mL solution while keeping HCl constant. Table 5 summarizes the concentrations of KF and HCl and corresponding pH values.

**Table 5:** Concentration of KF and HCl and the resulting pH values used for different batches (from A to T).

Batch	KF conc. / g mL^−1^	HCl conc. / g mL^−1^	pH (±0.2)	Batch	KF conc. / g mL^−1^	HCl conc. / g mL^−1^	pH (±0.2)

A	0.027	0.027	11.65	K	0.014	0.027	11.67
B	0.027	0.037	11.60	L	0.014	0.038	11.50
C	0.027	0.048	11.26	M	0.014	0.048	11.26
D	0.027	0.058	11.07	N	0.013	0.058	11.00
E	0.026	0.068	10.15	O	0.013	0.068	10.24
F	0.021	0.027	11.67	P	0.007	0.027	11.63
G	0.020	0.038	11.37	Q	0.007	0.038	11.48
H	0.020	0.048	11.27	R	0.007	0.048	11.11
I	0.020	0.058	10.80	S	0.007	0.058	10.70
J	0.020	0.068	10.05	T	0.007	0.068	9.97

### Procedure of the post-synthetic treatment with H_2_O_2_

Post-synthetic treatment by H_2_O_2_ was performed following the procedure reported in [26]. 200 mg of predried as-synthesized ETS-10 powder and 20 mL of 30 wt % aqueous H_2_O_2_ solution were loaded into a 90 mL PTFE reactor, which was inserted into a stainless steel autoclave and closed. The autoclave was put into the preheated to 423 K oven. The treatment was carried out in three autoclaves separately over 30 min, 45 min and 60 min. After the required duration, each autoclave was quenched with cold water down to room temperature and opened. The mixture in the reactor had a yellow color. Each sample was transferred to the centrifuge tube, washed with DDW and centrifuged at 3000 rpm three times. The centrifuged powders were finally dried in a convection oven at 423 K for 24 h. Afterwards, they were placed in a vacuum oven and further dried at 1 kPa and 373 K for 24 h to remove possible residual moisture. The post-synthetically treated samples were denoted as P-ETS-10/30, P-ETS-10/45, P-ETS-10/60 (where “P” stands for post-synthetically treated) according to corresponding treatment times of 30, 45 and 60 min, respectively.

### Calcination procedure

The catalyst P-ETS-10/60 underwent calcination (≈1 g) in a ceramic crucible. The calcination took place inside a NaberTherm^®^ P 300 muffle oven. The catalyst was not exposed to any thermal treatment prior to calcination. The calcination temperature of 873 K was reached with a ramp rate of 10 K min^−1^, after which the target temperature was held for 6 h. After this, the ramp rate for cooling was set at 10 K min^−1^. After room temperature was reached, the crucible was taken out of the muffle oven. The resulting material was labelled as C-P-ETS-10/60.

### Catalytic experiments

The catalytic reaction procedure was identical for all catalysts used in the transesterification reaction (CaO-800, CaO-900, CaO-1000, Na,K-ETS-10, P-ETS-10/60 and C-P-ETS-10/60). The reaction was carried out in sealed glass cylindrical tubes with a 7 mm inner diameter and 70 mm length resulting in 2.4 mL of available volume. A similar procedure was used in [20] to prevent methanol from evaporating during the course of reaction. The constituents of the reaction were added, after which the tube was quickly (<30 s) flame-sealed. 54 mg of a solid predried catalyst, 0.54 mL of triolein and 0.85 mL of methanol (measured at room temperature), resulting in a methanol/triolein molar ratio of 36:1, were used in all catalytic experiments.

After the glass tube was sealed, it was hand-shaken once during 15 s and loaded by ≈50 mm into the silicone oil bath preheated to 403 K; from this moment, the timing starts. The homogeneous heating of an oil bath (270 mL, 136 mm in diameter, 70 mm height) is provided by the 30 mm magnetic rod stirred using the IKA RCT basic magnetic stirrer at 600 rpm. An IKA ETS-D5 thermometer was used to control the temperature of the oil bath. After the desired reaction time, the tube was taken out of the bath, cooled down to room temperature and opened for further chromatographic analysis. Due to the initially poor miscibility of the triolein/methanol mixture, it is possible that after stopping the reaction, the mixture might remain still partly separated. This effect is expected to be less pronounced with the higher amount of methyl oleate formed. Thus, shortly before taking samples for analysis, the tube was again intensively shaken to minimize extraction of samples either from the triolein- or methanol-rich phases. After this, 10 μL of the reaction mixture was transferred to a centrifuge vial, preloaded with 3.2 mL of toluene and 0.013 mL dodecane. Toluene was used to promote dissolution of the reaction components, while dodecane was added as an internal standard for GC analysis. The solution, after the centrifugation for separation of the remaining catalyst, was transferred to another vial and analyzed by GC. To avoid the negative impact of sampling on the continuation of the reaction, the separate tubes were prepared (under the same contusions) for every reaction time studied.

Gas chromatographic analysis of the reaction mixtures used in the catalytic experiments was performed using a Shimadzu GC-2010 gas chromatograph equipped with a flame ionization detector (FID). For the separation of triolein and methyl oleate, an Rtx-Biodiesel TG capillary column (Restek, 10 m long, 0.32 mm inner diameter, 0.1 μm film thickness) was used. Nitrogen was used as the carrier gas at a flow rate of 2.14 mL min^−1^ with a purge flow rate of 3 mL min^−1^. The calibration procedure is described in section S2 of Supporting Information File 1.

### Powder X-ray diffraction

Powder X-ray diffraction (XRD) analysis of the CaO-based catalysts was performed using a Philips APD-1700 diffractometer with a monochromator and Cu anticathode in an angle diffraction span between 10 and 80° working at 55 mA and 40 kV. The crystallite sizes were calculated based on the data of the full width at diffraction line half maximum by using Scherrer's equation.

The XRD patterns of the titanosilicates were acquired on a Siemens D5000 diffractometer operating with Cu Kα radiation (λ = 0.15418 nm). The data were recorded in the 2θ range of 4–90° with a step of 0.05°. Approximately 100 mg of the solid sample was used in each experiment. The Miller indices of reflections obtained in the XRD patterns were analyzed using the “Match!” software.

### Scanning electron microscopy

Scanning electron microscopy (SEM) of the CaO-based catalysts was performed on a JEOL JSM-6460LV microscope at an accelerating voltage of 25 kV and different magnifications up to 100,000×. The samples were prepared for measurement by coating with gold nanolayers using the ion-sputtering chamber.

The SEM images of the titanosilicates were acquired using a LEO GEMINI 1530 device by Zeiss GmbH operating at an acceleration voltage between 5 and 30 kV. Approximately 1 mg of the solid samples was utilized for microscopy. The samples were analyzed on a carbon film on top of an aluminum stub. The samples were sputtered with a gold film using a BALZERS CED 030 sputter coater. The “Image J” software was used to analyze the microscopy images. SEM was carried out at the IMKM, Universität Leipzig.

Energy dispersive X-ray spectroscopy (EDX) was carried out with an Oxford Instruments (Model No. 7426) device with an energy resolution of 138 eV. The system utilizes the “INCA” software for creating EDX energy maps. EDX was utilized to determine the silicon (Si), titanium (Ti), oxygen (O), sodium (Na) and potassium (K) amount in atomic percent (atom %). Each determination of the atomic composition was based on four measurements by calculating the average atom %. The samples were sputtered with a carbon film.

### Transmission electron microscopy

Transmission electron microscopy (TEM) was carried out using a JEM-2100Plus instrument from JEOL operating at an accelerating voltage of 200 kV. The images were taken with a 4K CMOS camera from TVIPS. The TEM is equipped with a LaB_6_ cathode and high-resolution pole piece to achieve a point resolution in the TEM mode of 0.23 nm. The sample preparation was performed by grinding the sample in a mortar and pestle in ethanol and the dispersed particles were supported on a TEM lacey carbon grid.

### Nitrogen sorption experiments

N_2_ sorption isotherms were recorded at 77 K using a Micromeritics ASAP 2000 instrument. Before the measurements, the samples were degassed in vacuum (3 × 10^−11^ MPa) at 623 K for 8 h. The pore width distribution (PWD) in the mesopore range was calculated from the adsorption isotherms. The non-local density functional theory (NLDFT) kernel on silica (cylindrical pore, adsorption branch) in the “ASIQwin” software from Quantachrome Instruments was used for this purpose. In N_2_ adsorption experiments, 51 points were measured in the range of relative pressure between *P/P*_0_ = 0.05 (40 Torr) and *P/P*_0_ = 0.99 (760 Torr). The Brunauer–Emmett–Teller (BET) method was applied to calculate the specific surface area (*S*_BET_) from the adsorption branch of the isotherm in the relative pressure range of *P/P*_0_ = 0.06–0.30. The specific micropore volume (*V*_micro_) was determined using the *t*-plot method according to De Boer et al. [83] in the relative pressure range of *P/P*_0_ = 0.15–0.50. The total pore volume (*V*_Total_) was measured with the help of the Gurvich rule [84] at *P/P*_0_ = 0.99. The mesopore volume (*V*_meso_) was calculated by subtracting the *V*_Total_ by *V*_micro_.

### Mercury intrusion porosimetry

Mercury (Hg) intrusion porosimetry was carried out using a PASCAL 440 instrument reaching a maximum pressure of 400 MPa. Approximately 100 mg of a solid sample was used for characterization. The pore widths were calculated using the Washburn equation [85].

### Laser diffraction

Particle size analysis was carried out using the CILAS 1064 diffractometer. Approximately 200 mg were used for each measurement. The obscurity level was maintained at three. The measurement of a background signal was done prior to each experiment. An intense rinsing of the suspension with water was carried out after every measurement to remove residual particles from the previous measurements.

### Temperature-programmed desorption

Temperature-programmed desorption (TPD) experiments were carried out using an online Pfeiffer Vacuum quadrupole mass spectrometer (MS). The surface acid site and basic site density were determined using ammonia (NH_3_) and carbon dioxide (CO_2_) as probe molecules, respectively. After switching between probe gases, the instrument was flushed with the required gas for 24 h. The samples were flushed with 40 mL min^−1^ of (99 vol %) helium (He) from Air Liquide throughout the measurement process.

Prior to the TPD studies, ≈50 mg of the solid was placed into the reactor and pretreated at 573 K for 30 min. The sample was cooled down to different temperatures after pretreatment – to 363 K and 303 K in the case of NH_3_ and CO_2_, respectively. Loading with NH_3_/CO_2_ was carried out by purging the sample six times with doses of 1 mL min^−1^ for 1 min and then pulsing for 6 min. After purging, the samples were kept at that temperatures for 1.5 h to remove physisorbed NH_3_/CO_2_. For measuring the TPD profiles, the samples were heated at 10 K min^−1^ to 830 K, held for 30 min at 830 K and cooled to 303 K.

The desorbing gases were detected by a Pfeiffer Vacuum QME 200 mass spectrometer. For the detection of NH_3_, the mass fragment with *m*/*z* = 15 was recorded. For the NH_3_ oxidation products, N_2_O and NO mass fragments with *m*/*z* = 44 and 30, respectively, were recorded. The surface acid site densities were calculated from the amounts of desorbed NH_3_, N_2_O and NO under the assumption that desorbed N_2_O and NO were adsorbed as NH_3_ in the catalyst before they were oxidized. For the detection of CO_2_, the mass fragment with *m*/*z* = 44 was recorded. The surface basic site densities were calculated from the amount of desorbed CO_2_. The accuracy of the TPD-NH_3_/CO_2_ method was estimated to be ±5%.

### Differential thermal analysis

Differential thermal analysis was performed with 50 mg of the sample. The measurements were carried out on a Netzsch STA 409 TG/DTA cell coupled with a Balzers QMS 421 mass spectrometer equipped with a QMA 125 quadrupole analyzer system. The samples were analyzed in the temperature range of 283–1173 K with a heating rate of 278 K min^−1^ under an air stream.

### Optical emission spectrometry by inductively coupled plasma spectroscopy

Inductively coupled plasma optical emission spectrometry (ICP-OES) was used to determine elemental composition (wt %) of Ti, Na and K in the samples. The PerkinElmer OPTIMA 8000 ICP-OES instrument includes a CCD array detector that captures the full wavelength range at a high speed but does not allow simultaneous wavelength measurements. The sample introduction system used was a Cross Flow / Scott atomizer. Prior to analysis, the powdered samples (maximum 50 mg) were digested in a PTFE vessel in a mixture of 2 mL hydrofluoric acid, 3 mL nitric acid and 3 mL hydrochloric acid. The digestion occurred with the help of an Anton Parr Multiwave 3000 microwave oven having 8 XF100 rotors for chemical extraction. The microwave oven was kept at 1400 W, producing an internal temperature in the PTFE reactor of 448 K for 70 min. After digestion, 12 mL of boric acid were added for the complexation of hydrofluoric acid. The mixture was again treated for 10 min at 448 K in the microwave oven. After further microwave treatment, a clear solution was obtained and diluted with distilled deionized water to reach 50 mL for the ICP-OES analysis.

### ^1^H pulsed field gradient NMR

The diffusion experiments were performed using ^1^H NMR on a wide-bore 9.4 T Bruker BioSpin spectrometer. It was equipped with a home-built gradient unit, producing ultrahigh *z*-gradients of up to 35 T/m in a 7 mm (outer diameter) NMR sample compartment.

Around 180 mg of the material (C-P-ETS-10/60) was loaded into the glass tube. The latter was then connected to a custom-built vacuum system, and the samples were activated under high vacuum (10^−3^ Pa) at 400 K overnight. After cooling to room temperature, the sample was saturated under vacuum by injection of triolein (99 wt %, Sigma-Aldrich). The amount of liquid was chosen to be sufficient to cover the particles of the samples completely, i.e., oversaturated. This was done to ensure that all mesopores were completely filled. After loading, the NMR tubes were detached from the vacuum system and flame-sealed.

A 13-interval pulse sequence [86] was applied with the following parameters of the pulse sequence: τ = 2 ms, δ = 0.8 ms, and Δ = 20 ms. Here, τ is the spacing between the first two (π/2) pulses, δ is the effective duration of the gradient pulses, and Δ is the gradient pulse separation. The diffusion time *t*_d_ in this case is ≈20 ms. The signal accumulation was performed with a repetition of 5 times of *T*_1_, where *T*_1_ is the nuclear magnetic spin-lattice relaxation time of the component having the longest relaxation in the system.

The decrease of the measured NMR signal (Ψ) caused by diffusion for a system containing molecular ensembles (*p*_i_) with different diffusion coefficients (*D*_i_) and being in the slow (compared to the time of the experiment) diffusion exchange can be presented in the form [87]:

[2]
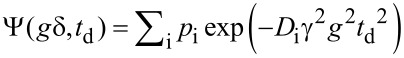


where γ is the gyromagnetic ratio of the nucleus that is observed, *g* and δ are the amplitude and the duration of the gradient pulses, respectively, *t*_d_ is the effective diffusion time (Δ−δ/6−τ/2 in the case of the 13-interval pulse sequence). Equation 2 gives a poly-exponential decay of the observed NMR signal, if i > 1.

### ^29^Si solid-state magic angle spinning NMR

The ^29^Si NMR spectra were recorded on a Bruker DRX-400 WB spectrometer (Bruker Biospin, Karlsruhe, Germany) with a 4 mm MAS BB/1H probe at a Larmor frequency of 79.49 MHz for ^29^Si and 400.15 MHz for protons, respectively. All of the spectra were acquired at a spinning speed of 12 kHz and at a temperature of 293 K. The spectra were referenced externally to tetramethylsilane (TMS).

The π/2 pulse was calibrated at a radio-frequency (rf) field strength of 100 kHz for ^1^H and 42 kHz for ^29^Si, respectively. For the CP experiments, 1024 scans were accumulated using an acquisition time of 25 ms. For the CP transfer, a linear rf field of 42 kHz for ^29^Si and a linearly ramped rf field ranging from 43 kHz to 62 kHz for protons within a contact time of 8 ms were applied. A recycle delay of 1 s was used. The spectral width was set to 32 kHz and the offset was placed at −50 ppm. During the acquisition, heteronuclear decoupling was achieved using a SW_f_-TPPM [88] at an rf frequency of 100 kHz.

For the HPDEC experiments, 256 scans were accumulated at an rf field strength of 42 kHz as a π/2 pulse on ^29^Si during an acquisition time of 25 ms. The spectra were obtained at a spinning speed of 12 kHz and a recycle delay of 40 s at a temperature of 293 K. The spectral width was set to 32 kHz and the offset was placed at −50 ppm. During the acquisition, heteronuclear decoupling was achieved using a SW_f_-TPPM at an rf frequency of 100 kHz.

### Hyperpolarized ^129^Xe NMR

The hyperpolarized (HP) ^129^Xe NMR spectra were recorded on a Bruker DRX-600 MHz spectrometer (Bruker Biospin, Karlsruhe, Germany) with a 5 mm TBI probe at a Larmor frequency of 166 MHz for ^129^Xe. The hyperpolarization of Xe nuclei was achieved by spin-exchange optical pumping (SEOP) using a home-built polarizer in continuous-flow mode as described elsewhere [89]. The gas mixture consisted of 1.1% Xe (natural isotopic portions, Air Liquide, purity: 99.998%), 27.5% N_2_ (Air Liquide, purity: 99.999%) and 71.4% He (Air Liquide, purity: 99.999%). The optical pumping cell contained rubidium (AlphaAesar, purity >99%). During the pumping process, the temperature was set to 423 K. The samples were dried for 14 h at 393 K and directly transferred to a standard 5 mm NMR tube equipped with a home-built gas insertion cap for continuous gas delivery.

For one-dimensional spectra, 32 scans were accumulated with an acquisition time of 60 ms, employing an rf field strength of 11 kHz for the ^129^Xe π/2 pulse and a recycle delay of 15 s. The spectral width was set to 42 kHz and the offset was placed at 30 ppm. The temperature calibration for the variable-temperature (VT) experiments was performed based on the proton chemical shift of a methanol calibration sample provided by Bruker. The temperature points were set to 190 K, 210 K, 230 K, 250 K, 270 K and 290 K.

The EXSY experiments were recorded at a temperature of 293 K where 8 scans were acquired for each of the 32 points in the F1 dimension. The mixing times were set to 1 ms and 100 ms, respectively. The rf field strength of the 90° pulse on ^129^Xe was 11 kHz, while the recycle delay was set to 7 s. The spectral width was set to 42 kHz and the offset was placed at 30 ppm in both dimensions.

For all spectra, the free gas signal was set to 0 ppm for referencing the chemical shifts. Due to alterations in filling height, packing density and polarization rate, the comparison of the absolute signal intensities between the samples cannot be made.

### Electron paramagnetic resonance

CW EPR measurements were carried out using a Bruker EMXmicro X-band spectrometer (≈9.41 GHz) equipped with a Bruker ER4119HS cylindrical cavity resonator. For low-temperature measurements, an Oxford ESR 900 flow cryostat was used. All spectra were recorded at *T* = 70 K with a modulation amplitude of 0.4 mT and a microwave power of 2.0 mW. Before and during the measurements, the samples were kept under ambient atmosphere.

## Supporting Information

File 1 Preparation and characterization of catalysts and performance of catalytic tests.
