# Supplementary material for: Synthesis of highly active ETS-10-based titanosilicate for heterogeneously catalyzed transesterification of triglycerides
Source: Beilstein J Nanotechnol. 2019 Oct 28;10:2039–61. doi: 10.3762/bjnano.10.200 (PMC6839565; doi:10.3762/bjnano.10.200)
Supplement: File 1 — Preparation and characterization of catalysts and performance of catalytic tests. [file Beilstein_J_Nanotechnol-10-2039-s001.pdf]

BEILSTEIN JOURNAL OF NANOTECHNOLOGY

Supporting Information

for

Synthesis of highly active ETS-10-based titanosilicate for heterogeneously catalyzed transesterification of triglycerides

Muhammad A. Zaheer, David Poppitz, Khavar Feyzullayeva, Marianne Wenzel, Jörg Matysik, Radomir Ljupkovic, Aleksandra Zarubica, Alexander A. Karavaev, Andreas Pöppl, Roger Gläser and Muslim Dvoyashkin

Beilstein J. Nanotechnol. **2019,** *10,* 2039–2061. doi:10.3762/bjnano.10.200

Preparation and characterization of catalysts and performance of catalytic tests

License and Terms: This is a supporting information file under the terms of the Creative Commons Attribution License (http://creativecommons.org/licenses/by/4.0). Please note that the reuse, redistribution and reproduction in particular requires that the authors and source are credited.
The license is subject to the *Beilstein Journal of Nanotechnology* terms and conditions: (https://www.beilstein-journals.org/bjnano)

Section Descriptions

S1: Graphical chart of the synthesis procedure of Na, K-ETS-10.

S2: Reaction tube. Calibration of the analytes for detection of triolein, diolein, monolein, methanol, and methyl oleate.

S3: Structure characterization of CaO-based catalysts.

S4: Textural data of CaO-900 and CaO-1000.

S5: Temperature programmed desorption of carbon dioxide (TPD-CO_2) of CaO-800, CaO-900 and CaO-1000.

S6: Diagram with values of KF- and HCl concentration used in preparation of different ETS-10 batches.

S7: Evaluation of the presence of different titanosilicate phases and quartz in the batch prepared for catalytic studies.

S8: Analysis of the energy dispersive X-ray spectroscopy (EDX) for identification of ETS-10, ETS-4, AM-1 and quartz phases.

S9: Transmission electron microscopy (TEM) images of Na, K-ETS-10 crystals.

S10: Estimated crystallinity values for Na, K-ETS-10, P-ETS-10/30, P-ETS10/45, P-ETS10/60 and C-P-ETS-10/60.

S11: Results of Hg-porosimetry of Na, K-ETS-10, P-ETS-10/60 and C-P-ETS-10/60.

S12: Hyperpolarized 2D-EXSY ^{129}Xe NMR experiments for probing exchange between pores of the crystals and outer space between the crystals.

S13: Schematical representation of the basic elements of the titanosilicate frameworks based on results of TPD, ^{29}Si MAS NMR, EPR and PFG NMR (including illustration of the changes in the framework resulting from treatment).

S1: Graphical chart of the synthesis procedure of Na,K-ETS-10

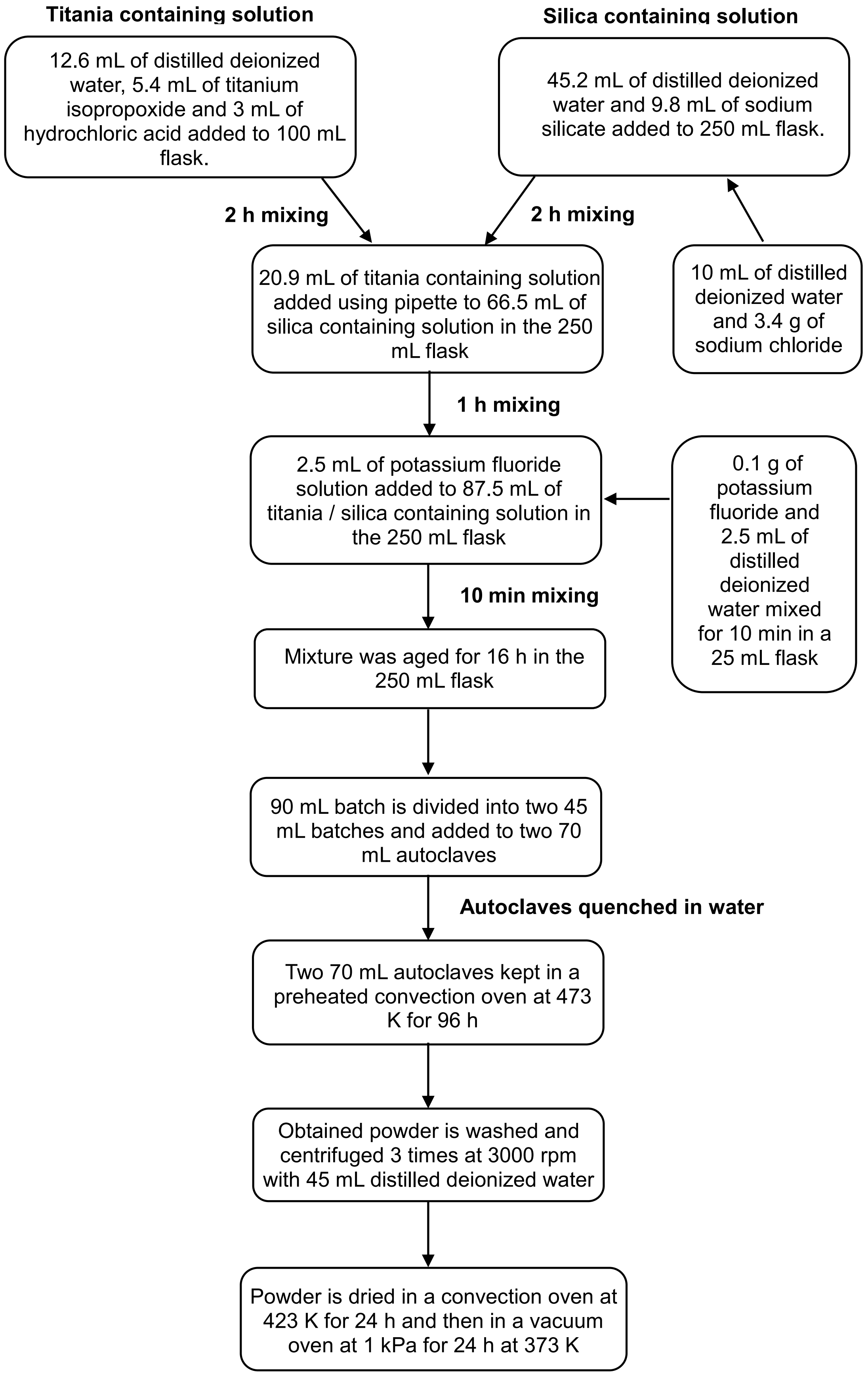

S2: Reaction tube. Calibration of the analytes for detection of triolein, diolein, monolein, methanol and methyl oleate

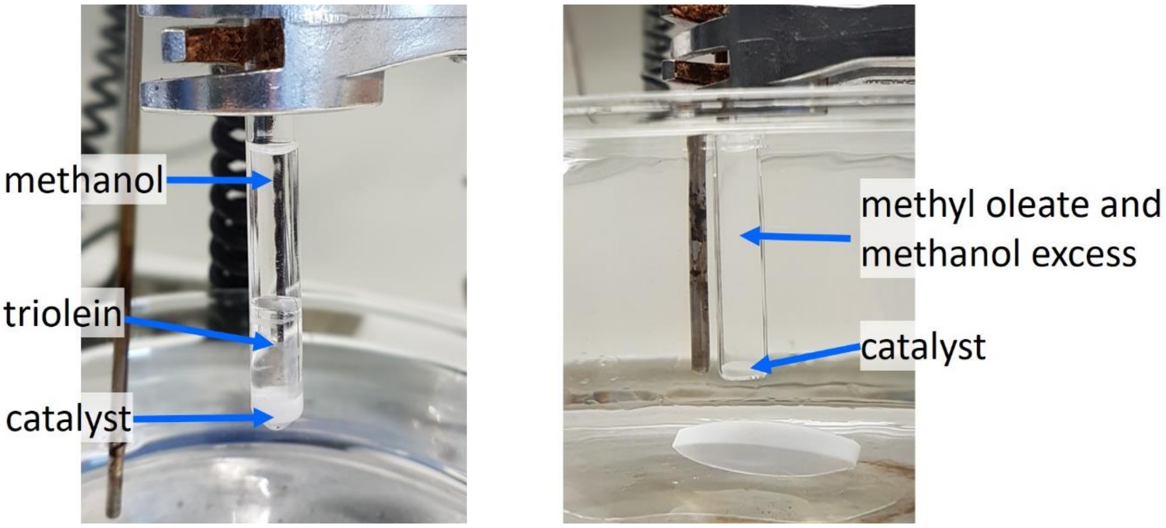

Figure S2.1: Pictures of the reaction tube before the reaction starts (left), and after the reaction is completed (right). The pictures are made with tubes outside (left) and inside (right) the oil bath.

The calibration of analytes was carried out using five equidistant concentration values in the following ranges: 0-12 mg mL^{-1} for triolein, 0.025-0.3 mg mL^{-1} for 1,3-diolein, 0.025-0.250 mg mL^{-1} for monoolein, 2-12 mg mL^{-1} for methyl oleate, and 0.5-2.5 mg mL^{-1} for methanol. The analytes were dissolved in a fixed amount of toluene and dodecane. Measured peak areas of corresponding analytes were normalized to the dodecane peak areas. The retention times recorded for analytes were 45.68 min (triolein), 16.55 min (1,3-diolein), 25.00 min (monoolein), 16.11 min (methyl oleate), 0.73 min (methanol), 1.03 min (toluene) and 5.89 min (dodecane). The obtained retention times were found to be consistent with reported values in literature [S1]. The

data were fitted using a linear function, from which the concentrations (in the units of mg mL^{-1}) of the analytes were obtained.

The values of the triolein conversion were calculated according to the following relation [S2]:

$$\text{Conversion of triolein (\%)} = 1 - \frac{[\text{triolein at a time } t \text{ (mg mL}^{-1})]}{[\text{triolein at a time } t = 0 \text{ (mg mL}^{-1})]} \times 100\ \%,$$

where [triolein at a time *t*] is a triolein concentration measured at a time *t* after the beginning of a reaction (*t=0*).

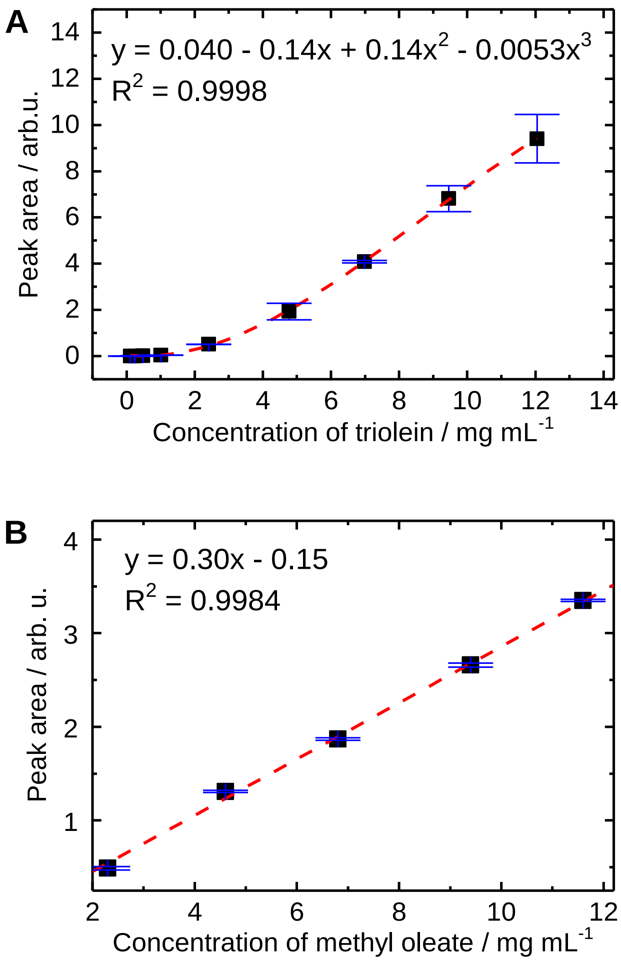

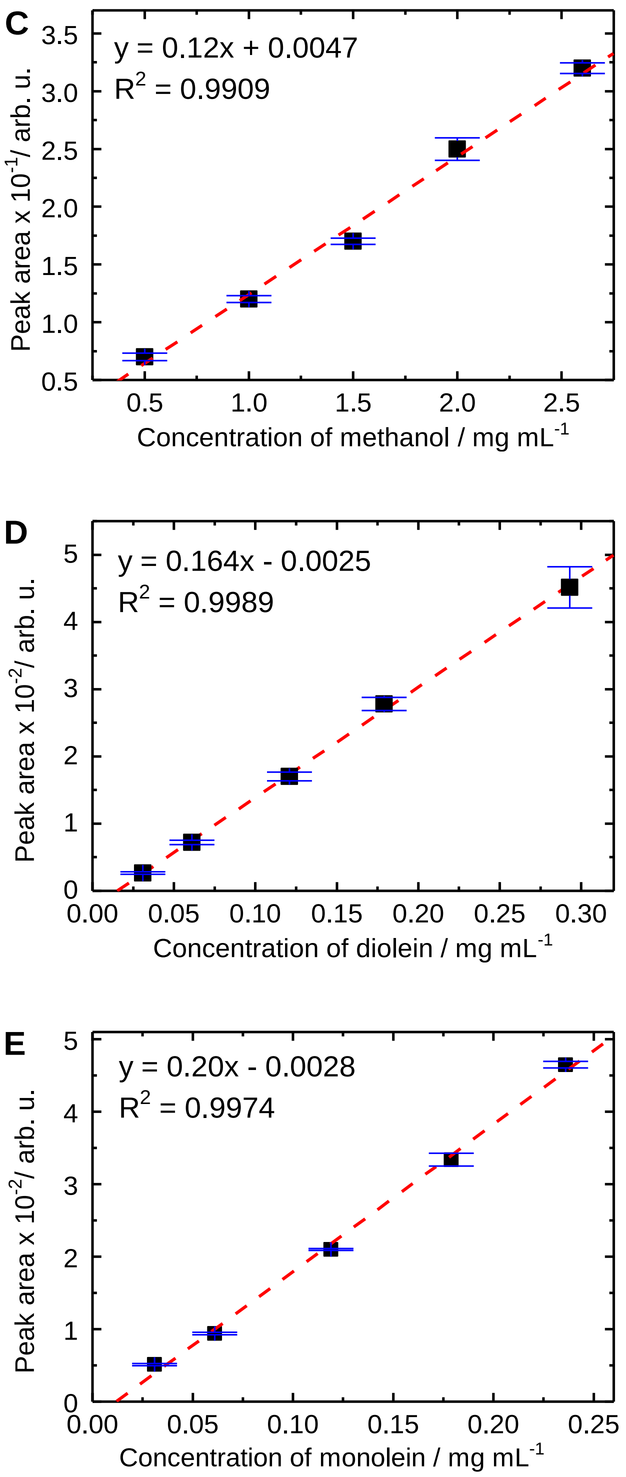

Figure S2.2: Calibration graphs of (A) triolein, (B) methyl oleate, (C) methanol, (D) diolein, and (E) monolein. Calibrations were done using the single-component triolein (0-12 mg mL^{-1}), monolein (0.025-0.250 mg mL^{-1}), 1,3-diolein (0.025-0.3 mg mL^{-1}), methyl oleate (2-12 mg mL^{-1}) and methanol (0.5-2.5 mg mL^{-1}).

S3: Structure characterization of CaO-based catalysts

The X-ray diffractograms of unmodified CaO-precursor and CaO catalysts calcined at different temperatures are shown in Figure S3.1. The diffractogram of the CaO precursor, in addition to the sharp characteristic diffraction of the CaO phase (peaks at 32.2°, 37.4°, 53.8°, 64.2° and 79.5°), reveals broader diffraction lines of the $Ca(OH)_2$ phase (28.7°, 34.1°, 47.1°, 50.8°, 59.4°, 62.6° and 71.8°) and the $CaCO_3$ phase (29.2° and 38.9°) [S3, S4]. With increasing calcination temperature, the carbonate and hydroxide phases become less present. However, the higher calcination temperature leads to sintering of CaO crystals resulting in larger sizes as it is evident from SEM images in Figure 3. This is also consistent with observed decrease of the specific surface area probed by N_2-sorption (Table S4).

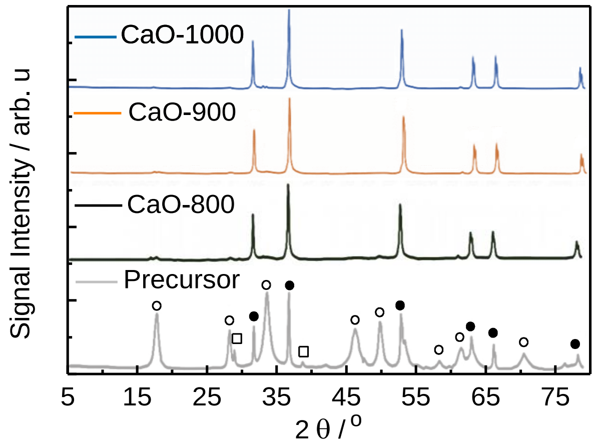

Figure S3.1: X-ray diffractograms of the CaO-based precursor and of that calcined at 1073 K (CaO-800), 1173 K (CaO-900) and 1273 K (CaO-1000). Circles and squares represent characteristic reflections of CaO (●), $Ca(OH)_2$ (○) and $CaCO_3$ (□).

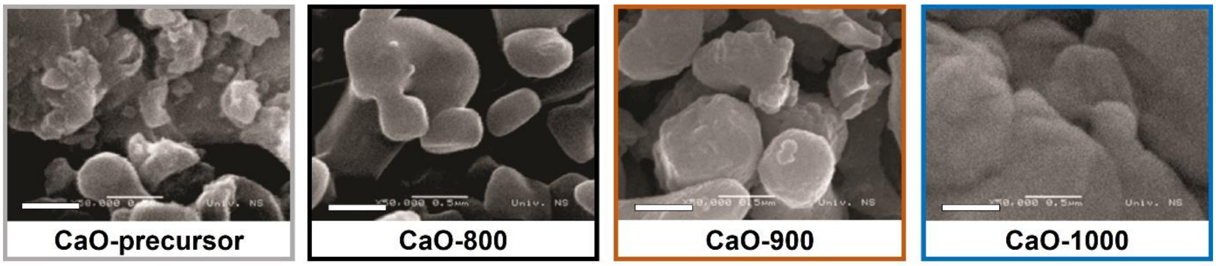

Figure S3.2: SEM images of the CaO precursor and of that calcined at 1073 K (CaO-800), 1173 K (CaO-900) and 1273 K (CaO-1000, from left to right). The scale bar represents 500 nm.

Basicity of the CaO catalysts needed for promotion of the transesterification process was confirmed by the TPD of CO_2 (Figure S3.3). Expectedly, all three catalysts revealed desorption above 640 K from the strong Brønsted basic sites with calculated densities of 315, 180 and 260 μmol g^{-1} in case of CaO-800, CaO-900, and CaO-1000. Table S4 of the ESI summarizes obtained specific surface areas and calculated intrinsic base adsorption capacities.

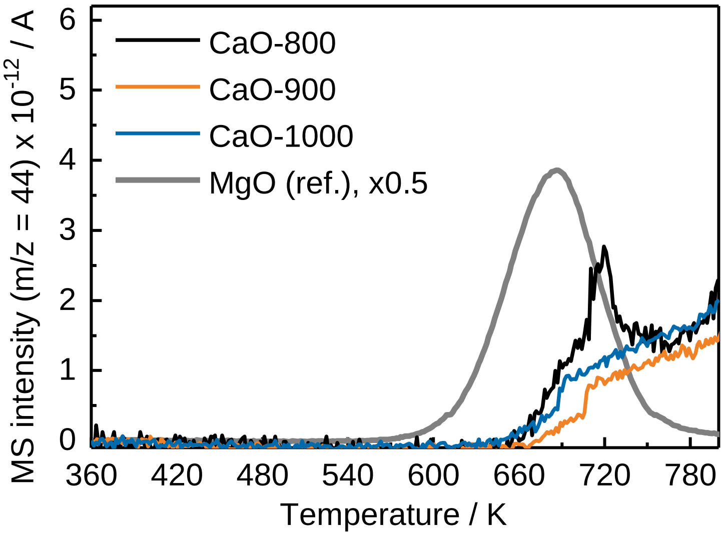

Figure S3.3: Temperature-programmed desorption of CO_2 from CaO catalysts calcined at 1073 K (CaO-800), 1173 K (CaO-900) and 1273 K (CaO-1000). The TPD curve of MgO was used as a reference.

S4: Textural data of CaO-900 and CaO-1000

Table S4: Textural characteristics of the CaO-900 and CaO-1000 from the results of N_2-sorption experiments

Material	Specific surface area (BET) / $m^2\ g^{-1}$	Specific micropore volume / $cm^3\ g^{-1}$	Specific mesopore volume / $cm^3\ g^{-1}$	Specific total pore volume / $cm^3\ g^{-1}$
CaO-900	6.1	0	0.022	0.022
CaO-1000	5.5	0	0.021	0.021

Note: Micropore volume was calculated by t-plot at a relative pressure range of P/Po = 0.08-0.2. Total pore volume was calculated at P/Po = 0.99 (Gurvich rule). The mesopore volume is calculated by subtracting the micropore volume from the total pore volume.

S5: Temperature programmed desorption of carbon dioxide (TPD-CO_2) of CaO-800, CaO-900 and CaO-1000

Table S5: TPD-CO_2 profile data from CaO catalysts calcined at 1073 K (CaO-800), 1173 K (CaO-900) and 1273 K (CaO-1000). The TPD curve of MgO was used as a reference.

Material	Surface basic site density B_{Total} / µmol g^{-1}	Specific Surface Area A_{BET}/ m^2 g^{-1}	Intrinsic base adsorption capacity* IB_{Total} / µmol m^{-2}
CaO-800	315	n.d.	n.d.
CaO-900	178	6.1	29
CaO-1000	261	5.5	48
MgO (Ref.)	959	15 [S5]	63.9

*Intrinsic basic adsorption capacity (µmol m^{-2}) = $\frac{\text{Surface basic site density (mmol g}^{-1}\text{)}}{\text{Specific Surface area (m}^{2}\text{ g}^{-1}\text{)}}$

The accuracy of this method can be estimated to be about ± 5 %.

n.d. – no available data.

S6: Diagram with values of KF- and HCl concentration used in preparation of different ETS-10 batches

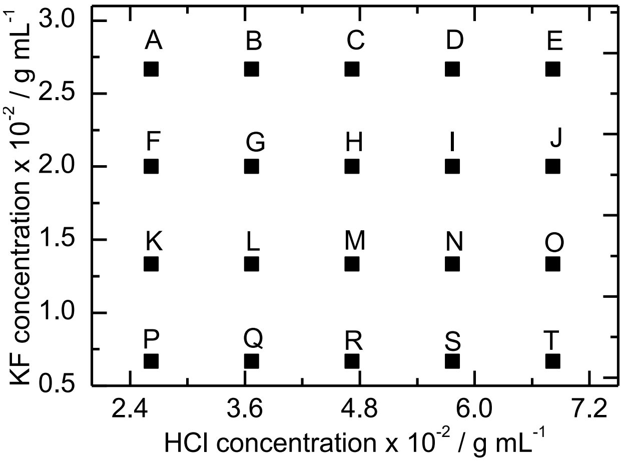

Figure S6.1: Crystallization graph showing the order in labelling of batches synthesized with varied concentration (g mL^{-1}) of HCl and KF.

The batches of ETS-10 prepared by varying KF- and HCl concentration. The obtained X-ray diffractograms were fitted using the reference ones and analysed using the Rietveld refinement. The obtained Bragg values [S6] are summarized in Table S6. The Reitveld refinement measurements were performed with the help of Match! and Full Prof software. The threshold value of 5% was not achieved. Thus, for evaluation of the presence of possible alternative phases the SEM images were used.

Table S6: Percentage of titanosilicates and quartz phase in each batch according to Rietveld refinement.

Batch	ETS-10 / %	ETS-4 / %	AM-1 / %	Quartz / %	Bragg R_B / %
A	37.7	32.6	29.0	0.7	41.4
B	61.9	7.0	31.1	0.0	57.9
C	78.4	5.2	16.4	0.1	57.9
D	40.2	1.5	57.1	1.1	47.3
E	8.1	1.6	87.2	3.0	59.5
F	50.4	30.4	18.1	1.1	39.5
G	57.7	7.6	34.3	0.4	61.9
H	33.6	3.3	62.5	0.6	48.3
I	8.1	1.5	88.3	2.1	51.9
J	5.9	0.9	92.4	0.8	39.1
K	13.6	8.0	77.1	1.3	47.1
L	64.0	7.0	28.3	0.7	58.0
M	46.1	2.1	51.2	0.6	56.4
N	1.1	0.2	93.4	5.3	41.3
O	6.5	2.1	90.8	0.6	47.4
P	8.3	5.8	85.3	0.6	44.9
Q	26.3	2.8	70.4	0.5	36.8
R	8.5	0.5	90.9	0.2	36.9
S	4.0	0.5	85.6	9.9	40.3
T	1.2	0.5	97.8	0.5	22.9

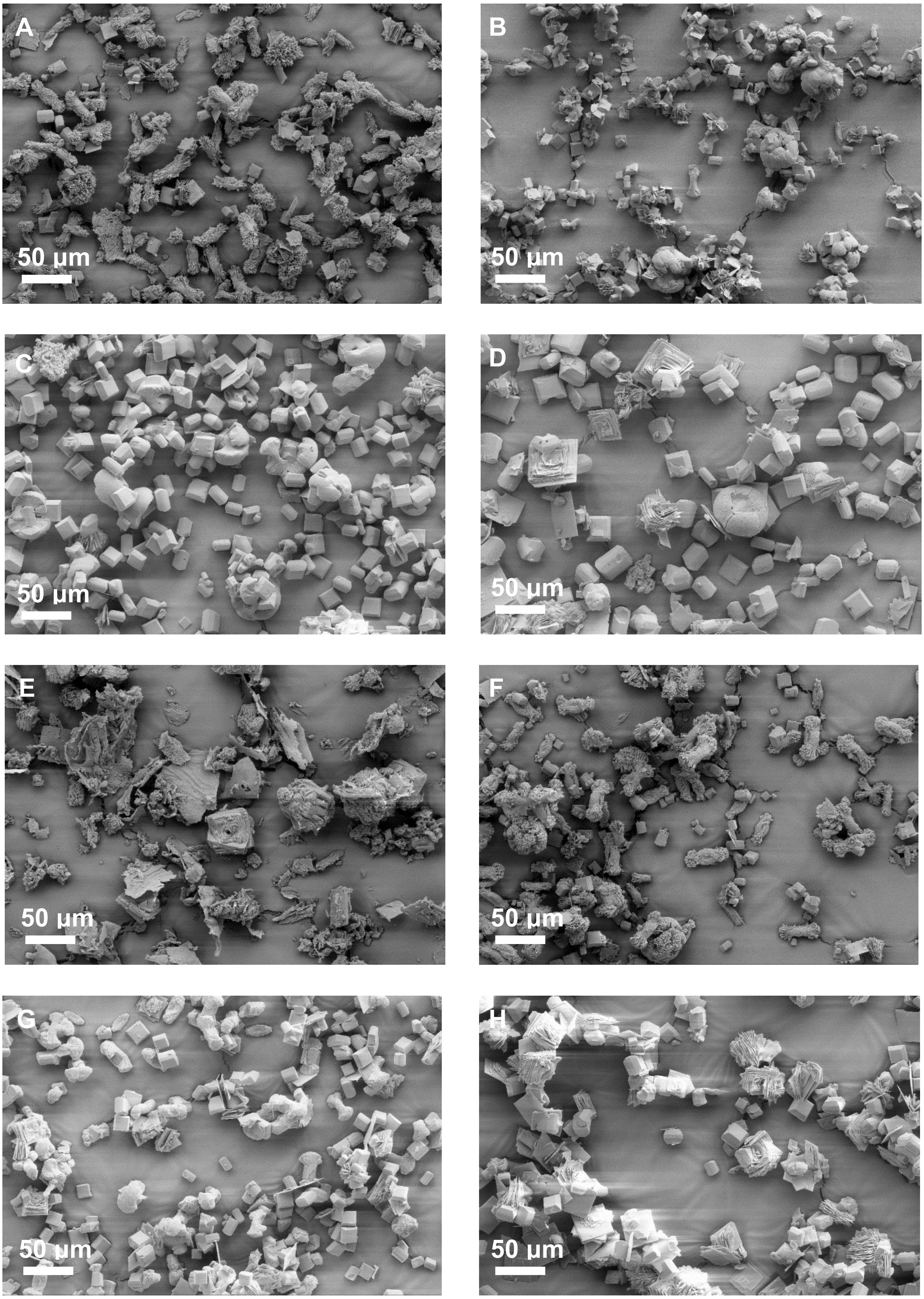
A
50 µm
B
50 µm
C
50 µm
D
50 µm
E
50 µm
F
50 µm
G
50 µm
H
50 µm

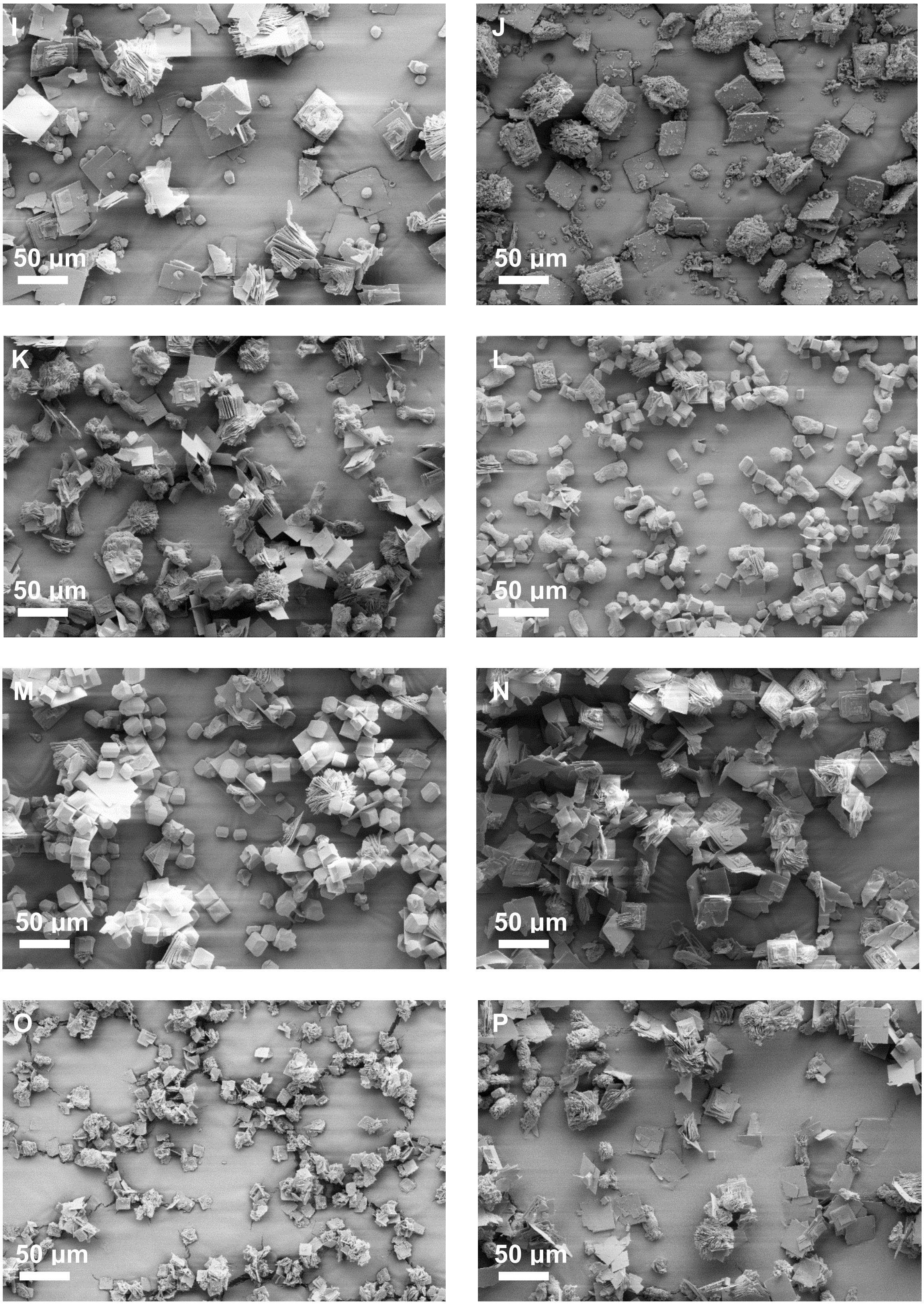
I
50 µm
J
50 µm
K
50 µm
L
50 µm
M
50 µm
N
50 µm
O
50 µm
P
50 µm

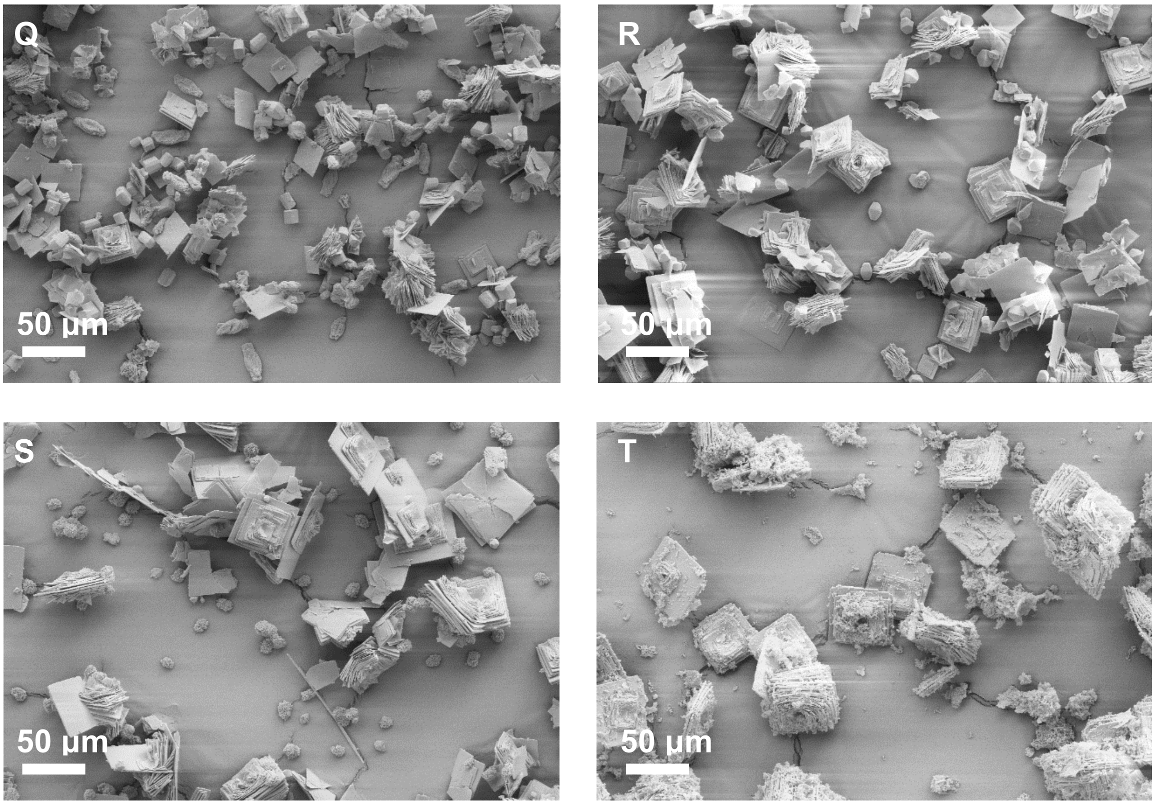

Figure S6.2: SEM micrographs of ETS-10 batches prepared with varied concentration (g mL^{-1}) of HCl and KF as shown in crystallization graph in Figure S6.1. The symbols in the top left corner match with the batch name of Table S6.

S7: Evaluation of the presence of different titanosilicate phases and quartz in the batch prepared for catalytic studies

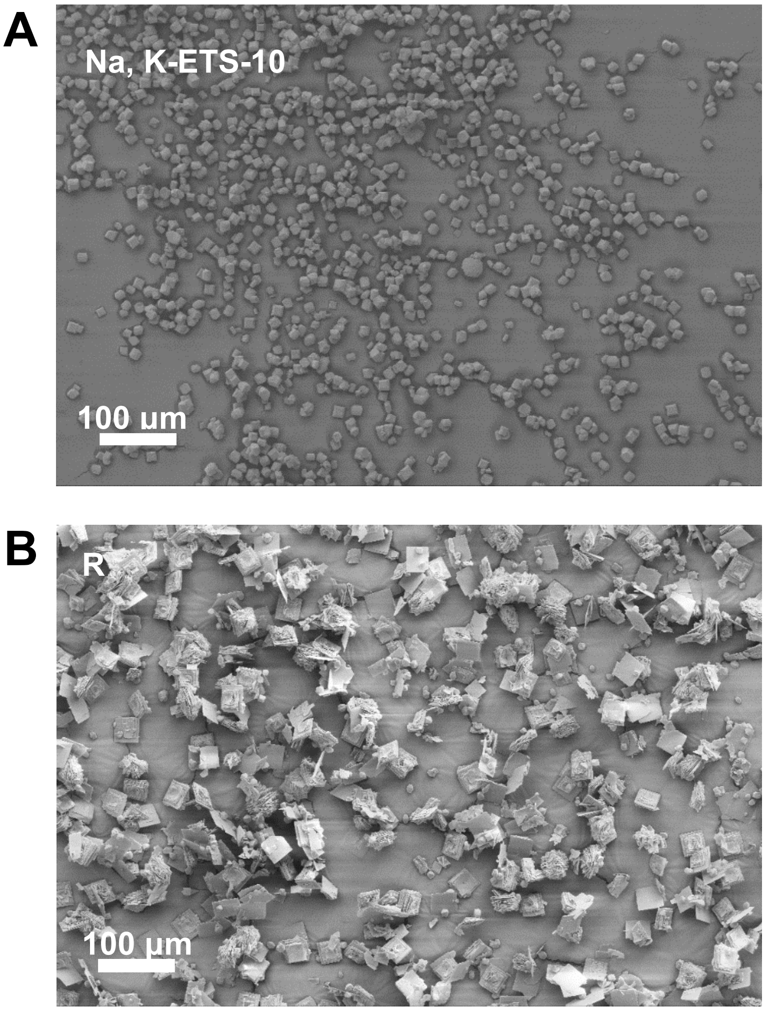

Figure S7.1: SEM micrographs of the Na,K-ETS-10 (A) and of the batch "R" (B).

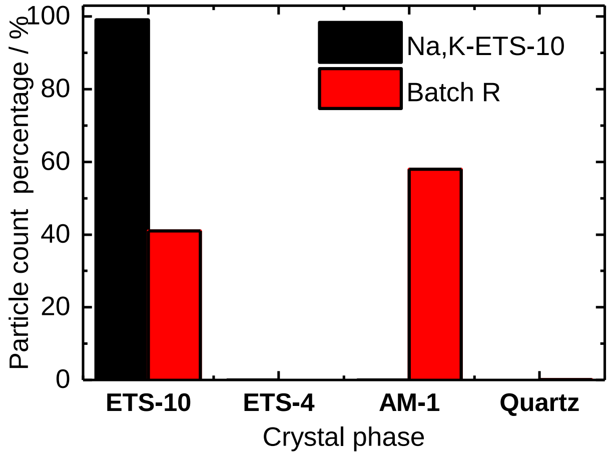

Figure S7.2: Particle count percentage of ETS-10, ETS-4, AM-1 and quartz crystals obtained from visual observation of SEM micrographs (Figure S7.1). The total particle count for Na,K-ETS-10 was 965 and for batch “R” was 508.

S8: Analysis of the energy dispersive X-ray spectroscopy (EDX) for identification of ETS-10, ETS-4, AM-1 and quartz phases

Table S8: EDX result of SEM micrographs of ETS-10, ETS-4, AM-1 and quartz in Figure S7.1.

Sample	*O*/ at.-%	*Na* / at.-%	*K* / at.-%	*Si* / at.-%	*Ti* / at.-%	O/Si ratio	Si/Ti ratio	Average
ETS-10 (1)	69.7	6.6	1.6	19.0	3.1	-	6.1	5.9
ETS-10 (2)	65.2	6.0	2.4	22.4	4.0	-	5.7	
ETS-10 (3)	71.6	6.2	1.4	18.0	2.9	-	6.3	
ETS-10 (4)	65.9	5.8	1.8	22.4	4.1	-	5.4	
ETS-4 (1)	65.6	6.9	3.3	18.0	6.2	-	2.9	2.8
ETS-4 (2)	68.3	7.6	2.7	16.2	5.2	-	3.1	
ETS-4 (3)	63.4	6.0	3.9	18.9	7.8	-	2.4	
ETS-4 (4)	67.0	7.4	2.9	16.7	6.0	-	2.8	
AM-1	71.2	8.4	-	17.1	3.4	-	5.0	4.2

(1)								
AM-1 (2)	70.5	9.1	-	17.0	3.5	-	4.8	
AM-1 (3)	62.3	7.6	-	23.6	6.1	-	3.9	
AM-1 (4)	66.9	7.8	-	19.3	6.1	-	3.2	
Quartz (1)	73.7	0.5	-	25.8	-	2.9	-	2.4
Quartz (2)	67.1	5.2	0.9	24.04	2.8	2.8	-	
Quartz (3)	60.5	1.2	0.6	36.9	0.8	1.6	-	
Quartz (4)	69.9	-	-	30.1	-	2.3	-	

Note: The numbers from (1-4) indicate the number of measurements performed on each individual particle of ETS-10, ETS-4, AM-1 and quartz.

S9: Transmission electron microscopy (TEM) images of Na,K-ETS-10 crystals

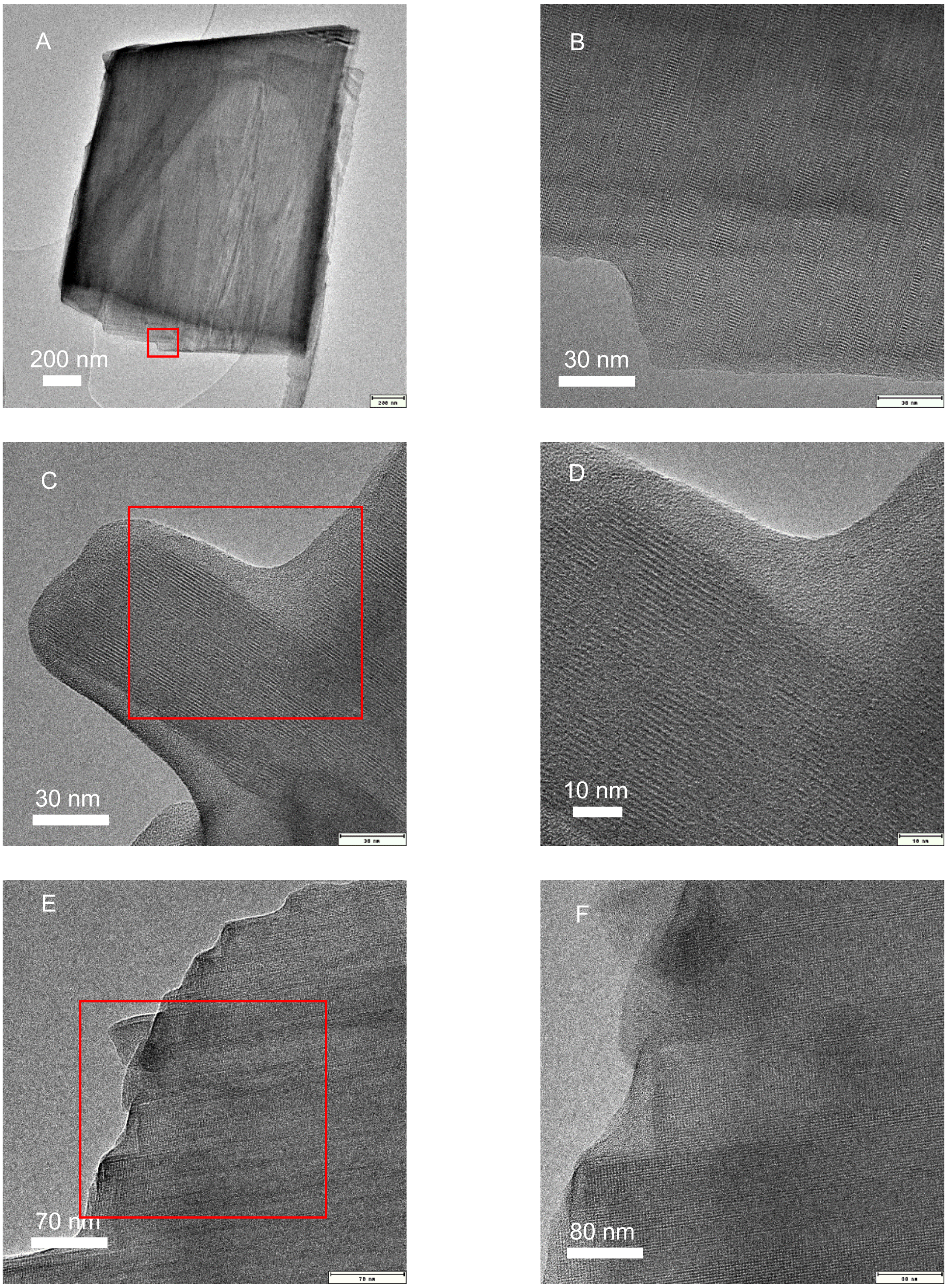

Figure S9: TEM micrographs of different Na,K-ETS-10 crystals (A, C, E). B, D, and E represent respective magnified areas.

S10: Estimated crystallinity values for Na,K-ETS-10, P-ETS-10/30, P-ETS10/45, P-ETS10/60 and C-P-ETS-10/60

Table. S10: Relative crystallinity (%) obtained for Na,K-ETS-10, P-ETS-10/30, P-ETS10/45, P-ETS10/60 and C-P-ETS-10/60 form the XRD data.

**Sample Name**	**Relative crystallinity[a] *RC* / %**
Na,K-ETS-10	100
P-ETS-10/30	74
P-ETS-10/45	65
P-ETS-10/60	77
C-P-ETS-10/60	100

[a]The relative crystallinity (%) were calculated using the standard method described in Ref. [S7, S8]. This was done firstly by calculating the area under the peak for 2θ = 6°, 24.7^o, 25.8^o, 27.2^o, 30° (d-spacing 1.46, 0.36, 0.34, 0.33 and 0.30 nm). Then followed by comparing calculations with Na,K-ETS-10 (which is arbitrarily assumed to have 100 % crystallinity) to determine relative crystallinity of P-ETS-10/30, P-ETS-10/45, P-ETS-10/60 and C-P-ETS-10/60.

S11: Results of Hg-porosimetry of Na,K-ETS-10, P-ETS-10/60 and C-P-ETS-10/60

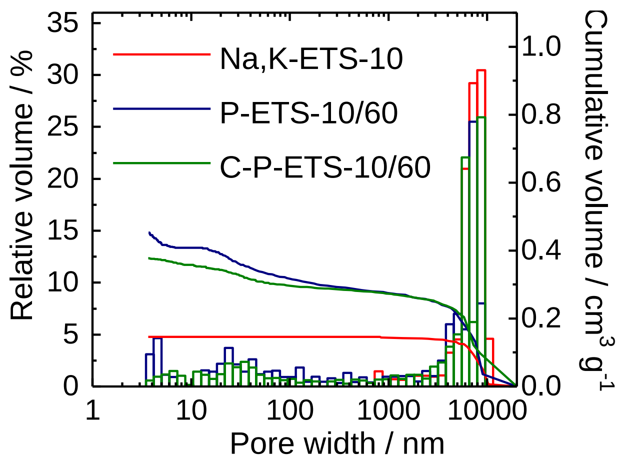

Figure S11: Hg-intrusion data for Na,K-ETS-10 (red), P-ETS-10/60 (blue) and C-P-ETS-10/60 (green). The bars represent relative volumes (left scale). The lines represent cumulative volumes (right scale)

S12: Hyperpolarized 2D-EXSY ^{129}Xe NMR experiments for probing exchange between pores of the crystals and outer space between the crystals

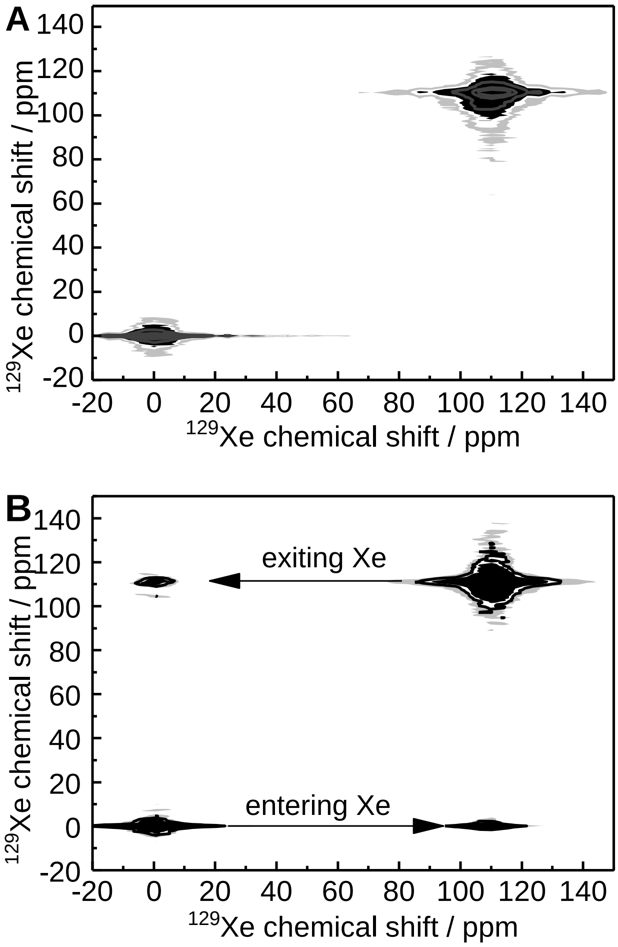

Figure S12: 2D-EXSY spectra of ^{129}Xe for Na,K-ETS-10 acquired at mixing times of 1 ms (A) with no observed cross-peaks and 100 ms (B) with visible cross-peaks caused by exchange between the intracrystalline pore space and the intercrystalline voids.

S13: Schematic representation of the elements of the titanosilicate frameworks based on results of TPD, ^{29}Si MAS NMR, EPR and PFG NMR

The following notation and colour coding has been used:

- **$Na^{(I)}$-$Na^{(V)}$** – Na cations according to their coordination in the framework;
- Ti, Ti–O–Ti – titanium nanowires
- –O–H – isolated, vicinal and strong interactive vicinal hydroxyl groups
- –Si–O• and –Si–O–O• – non bridging oxygen (NBO) and peroxy radicals
- **X** – the pore is not accessible for triolein
- **O** – Brønsted basic sites not accessible for triolein
- ✓ – the pore is accessible for triolein
- **O** – Brønsted basic sites accessible for triolein
- Si–O–Si – siloxane groups (appeared after calcination)
- Si–O–Si – siloxane groups forming the 5-member, 7-member and 12-member rings
- **N1** – sites seen in the low-temperature region of TPD-NH_3
- **N2** – sites seen in the high-temperature region of TPD-NH_3
- **C1** – sites seen in the low-temperature region of TPD-CO_2
- **C2** – sites seen in the high-temperature region of TPD-CO_2
- **A1-A4** and **B1-B2** – Si-atoms as seen by the ^{29}Si MAS NMR

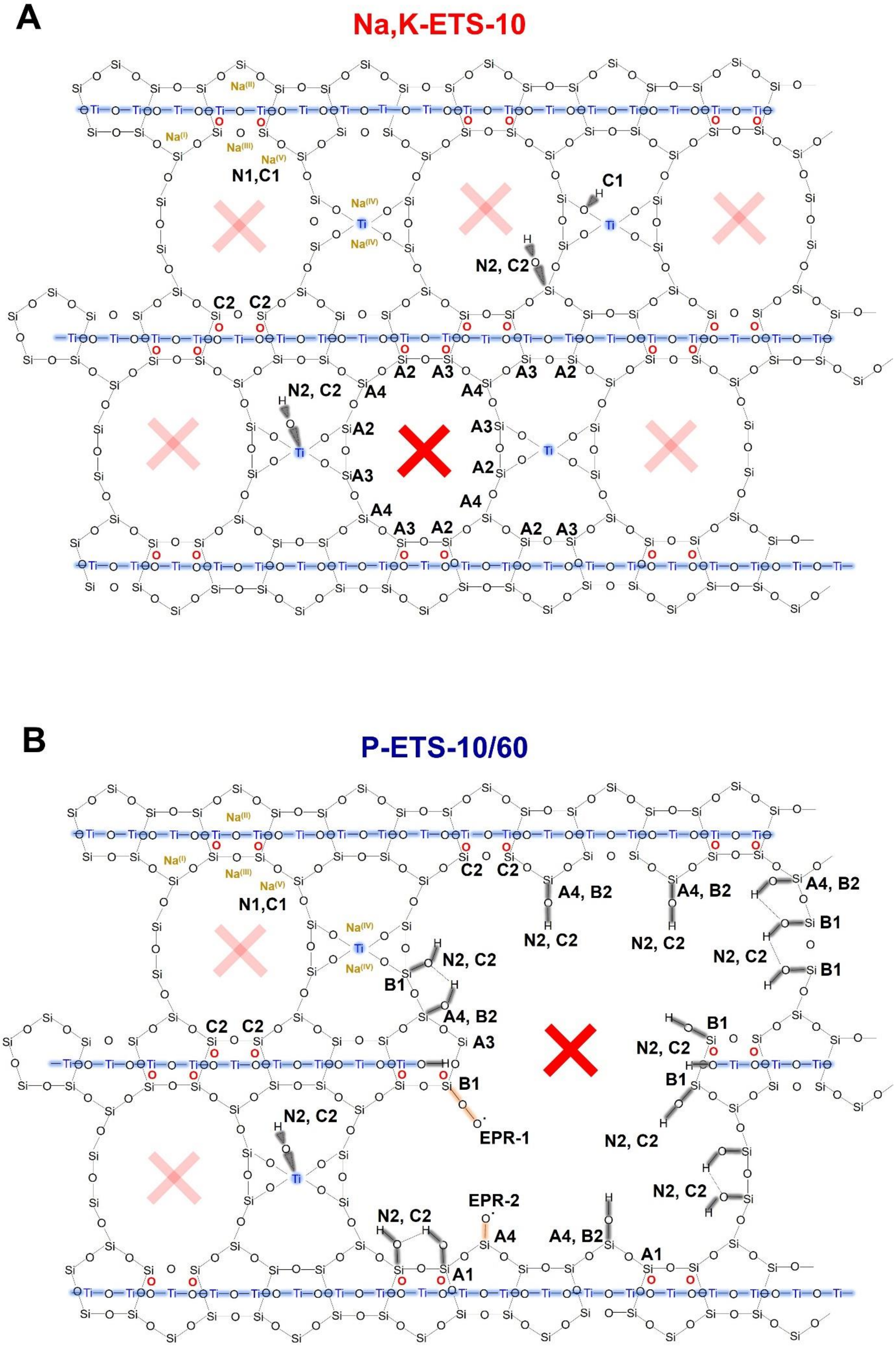
A
Na,K-ETS-10
N1,C1
C1
N2, C2
C2
A2
A3
A4
B
P-ETS-10/60
A4, B2
B1
A1
EPR-1
EPR-2

C

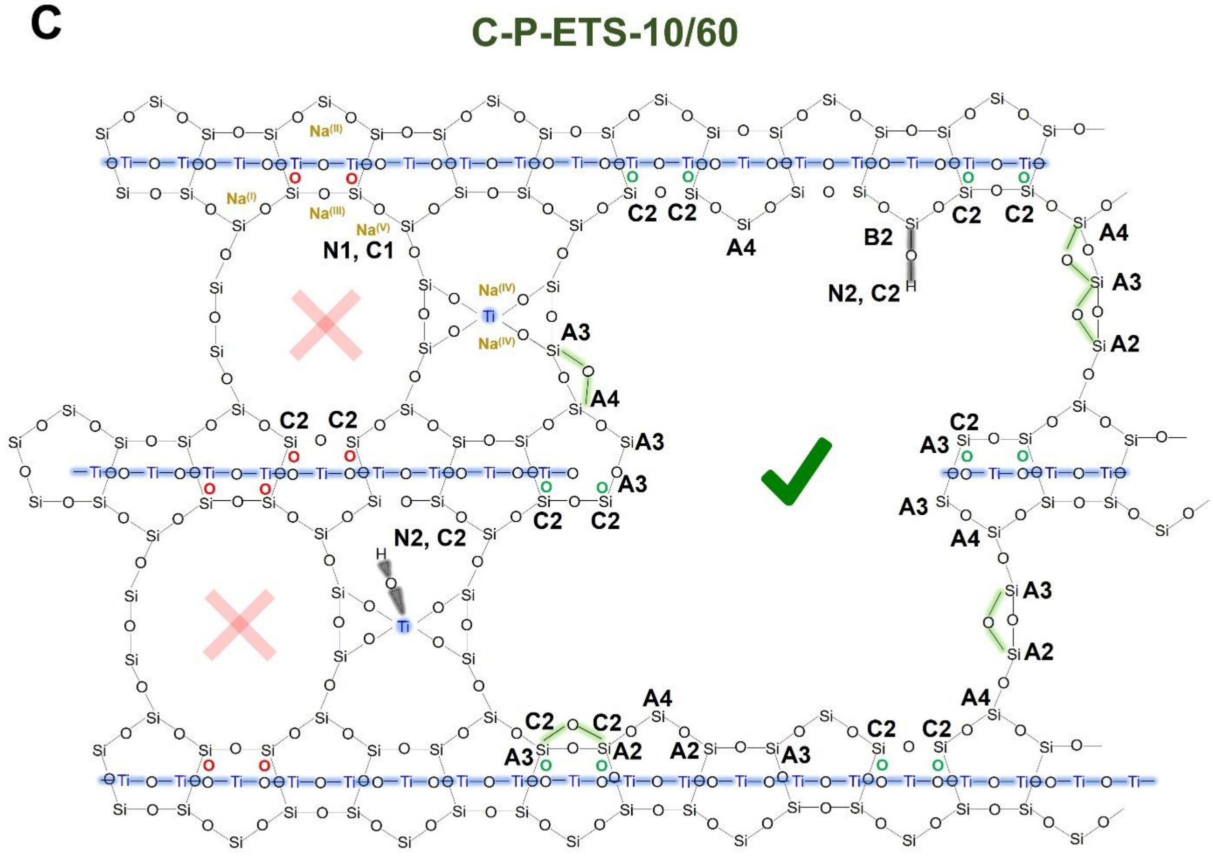

Figure S13: Schematic illustrations of (A) Na,K-ETS-10, (B) P-ETS-10/60 and (C) C-P-ETS-10/60. The illustrations are based on the results of TPD-NH_3/CO_2 (Figure 12), ^{29}Si MAS NMR (Figure 13), EPR (Figure 14), and PFG NMR (Figure 16).

References

S1. Marulanda, V. F.; Anitescu, G.; Tavlarides, L. L. *J. Supercrit. Fluids* **2010,** *54*, 53–60.

S2. Levenspiel, O. *Chemical reaction engineering*, 3rd ed.; Wiley: New York, 1999.

S3. Granados, M. L.; Poves, M. Z.; Alonso, D. M.; Mariscal, R.; Galisteo, F. C.; Moreno-Tost, R.; Santamaría, J.; Fierro, J.L.G. *Appl. Catal., B* **2007,** *73*, 317–326.

S4. Tang, Y.; Xu, J.; Zhang, J.; Lu, Y. *J. Cleaner Prod.* **2013,** *42*, 198–203.

S5. Ahmed, S.; Iqbal, A. *Global Challenges* **2018,** *2*, 1800056.

S6. David, W. I. F. *J. Res. Natl. Inst. Stand. Technol.* **2004,** *109*, 107–123.

S7. Lv, L.; Su, F.; Zhao, X. S. *Microporous Mesoporous Mater.* **2004,** *76*, 113–122.

S8. Stepto, R. F. T. *Polymer networks. Principles of their formation, structure and properties*, 1. ed.; Blackie Academic & Professional: London, 1998.
